# Synergistic Effects of Green Nanoparticles on Antitumor Drug Efficacy in Hepatocellular Cancer

**DOI:** 10.3390/biomedicines13030641

**Published:** 2025-03-05

**Authors:** Mirela Claudia Rîmbu, Liliana Popescu, Mirela Mihăilă, Roxana Colette Sandulovici, Daniel Cord, Carmen-Marinela Mihăilescu, Mona Luciana Gălățanu, Mariana Panțuroiu, Carmen-Elisabeta Manea, Adina Boldeiu, Oana Brîncoveanu, Mihaela Savin, Alexandru Grigoroiu, Florin Dan Ungureanu, Emilia Amzoiu, Mariana Popescu, Elena Truță

**Affiliations:** 1 Medical Doctoral School, Titu Maiorescu University of Bucharest, 040317 Bucharest, Romania; mirela.rimbu@prof.utm.ro (M.C.R.); fdungureanu@gmail.com (F.D.U.); 2 Faculty of Pharmacy, Titu Maiorescu University, Gheorghe Sincai Blv. 16, 040314 Bucharest, Romania; liliana.popescu2024@prof.utm.ro (L.P.); mirela.mihaila@virology.ro (M.M.); roxana.sandulovici@prof.utm.ro (R.C.S.); luciana.galatanu@prof.utm.ro (M.L.G.); mariana.panturoiu@prof.utm.ro (M.P.); carmen.manea@prof.utm.ro (C.-E.M.); mari.popescu@yahoo.com (M.P.); helen_truta@yahoo.com (E.T.); 3 Ștefan S. Nicolau Institute of Virology, 285 Mihai Bravu Street, 030304 Bucharest, Romania; 4 National Institute for Research and Development in Microtechnologies (IMT Bucharest), 072996 Bucharest, Romania; adina.boldeiu@imt.ro (A.B.); oana.brincoveanu24@gmail.com (O.B.); mihaela.savin@imt.ro (M.S.); alexandru.grigoroiu@imt.ro (A.G.); 5 Faculty of Pharmacy, University of Medicine and Pharmacy of Craiova, 200349 Craiova, Romania; emanro2002@yahoo.com; 6 Horia Hulubei National Institute for R&D in Physics and Nuclear Engineering (IFIN-HH), 30 Reactorului Street, 077125 Măgurele, Romania

**Keywords:** antitumoral, green nanoparticles, biosynthesis, synergistic effect, pharmacology, nanomedicine, liver cancer, plant extracts, cytotoxicity, *Clematis vitalba*, *Melissa officinalis*

## Abstract

**Background/Objectives**: Cancer remains one of the leading causes of mortality worldwide. Despite significant advancements in treatment strategies and drug development, survival rates remain low and the adverse effects of conventional therapies severely impact patients’ quality of life. This study evaluates the therapeutic potential of plant-derived extracts in hepatocellular carcinoma treatment, with a focus on minimizing side effects while enhancing efficacy. **Methods**: This research investigates the in vitro synergistic effect of silver bio-nanoparticles synthesized from *Clematis vitalba*, *Melissa officinalis*, and *Taraxacum officinale* extracts (*Clematis vitalbae extractum*—CVE, *Melissae extractum*—ME, *Taraxaci extractum*—TE) in combination with liver cancer drugs, sunitinib (SNTB) and imatinib (IMTB), on HepG2 (human hepatocellular carcinoma) and HUVEC (human umbilical vein endothelial) cell lines. The silver nanoparticles (AgNPs) were characterized using UV-Vis spectroscopy, dynamic light scattering (DLS), zeta potential analysis, and scanning electron microscopy (SEM). The antitumor effects were evaluated through cell viability assays after 24 and 48 h of exposure, with additional cytotoxicity tests on HUVEC cells. **Results**: Results indicated that *Melissa officinalis*-derived silver nanoparticles (ME AgNPs) and *Clematis vitalba* extract with silver nanoparticles (CVE AgNPs) significantly reduced HepG2 cell viability. Their efficacy improved when combined with conventional therapies (SNTB + ME AgNPs 1:1 vs. SNTB: 20.01% vs. 25.73%, *p* = 0.002; IMTB + ME AgNPs 1:1 vs. IMTB: 17.80% vs. 18.08%, *p* = 0.036; SNTB + CVE AgNPs 1:1 vs. SNTB: 18.73% vs. 25.73%, *p* = 0.000; SNTB + CVE AgNPs 1:2 vs. SNTB: 26.62% vs. 41.00%, *p* = 0.018; IMTB + CVE AgNPs 1:1 vs. IMTB: 12.99% vs. 18.08%, *p* = 0.001). *Taraxacum* extract exhibited similar cytotoxicity to its nanoparticle formulation but did not exceed the efficacy of the extract alone at 24 h. Selectivity index assessments confirmed that AgNPs-based formulations significantly improve cytotoxicity and selectivity to HepG2 cells. Among the tested extracts, CVE demonstrated the strongest antitumor effect, enhancing the efficacy of synthetic drugs (CI < 1). SNTB + TE AgNPs (5% EtOH) also demonstrated consistent synergy at high doses, while SNTB + CVE AgNPs provided broad-range synergy, making it suitable for dose-escalation strategies. **Conclusions**: These findings underscore the potential of nanoparticle-based formulations in combination therapies with targeted kinase inhibitors such as sunitinib and imatinib. Future research should focus on in vivo validation and clinical trials to confirm these findings.

## 1. Introduction

Cancer, the second leading cause of mortality worldwide after cardiovascular diseases in developed nations, is characterized by the uncontrolled multiplication and spread of abnormal cells in the body. Today, cancer remains a significant challenge for pharmacology, despite major advances in treatment and drug development that have allowed more people to live longer, reaching ages where malignant tumors become common [1].

Although both benign and malignant tumors involve uncontrolled cell proliferation, malignant tumors are distinguished by their capacity for dedifferentiation, invasiveness, and ability to metastasize. This behavior reflects altered gene expression patterns in cancer cells, resulting from acquired or inherited mutations [1].

Current anticancer drugs include hormones, protein kinase inhibitors, monoclonal antibodies, miscellaneous agents, and antiproliferative agents that damage DNA and trigger cell apoptosis by affecting cell division. Natural plant derivatives affecting microtubule function, including taxanes, *Vinca* alkaloids, and camptothecins, represent a specific therapeutic synthesis and have inspired the study of other plant-derived products as important sources of antitumor compounds [1]. Among these are alkaloids, phenolics, carotenoids, and flavonoids, which are highly researched for their wide range of medicinal properties, including antitumor activity on various cancer cell types [2,3,4,5].

The medical importance of plant-derived compounds is well recognized in oncology, especially as an alternative to the limitations of conventional drug therapies, which can involve severe toxicity, side effects, or even inefficacy due to the emergence of multidrug resistance [6]. Several studies have highlighted the spectrophotometric evaluation of the polyphenolic profile, the antioxidant activity, and the antiproliferative effects of dandelion extracts (*Taraxacum officinale*), noting their potential to inhibit the growth of various tumor cells in a dose-dependent manner [7,8,9]. However, the anticancer activity of dandelion remains anecdotal, requiring solid comparative studies with synthetic anticancer agents to substantiate its antitumor effects. Dandelion extracts have shown in previous studies the ability to exert antiangiogenesis effects both in vivo and in vitro on hepatocellular carcinoma by suppressing the expression of VEGF and HIF-1α [10]. Additionally, they can inhibit proliferation, induce apoptosis, and cause cell cycle arrest in hepatocellular carcinoma cell lines such as HepG2 and Hs7, possibly through immunomodulation by increasing CD4+ T cell ratios and T cell infiltration in tumor tissues [11]. Investigations into the antiproliferative activity of methanolic extracts of dandelion root on cell viability of HepG2, MCF7, HCT116, and normal Hs27 cells have shown that they can activate and control the AMP protein kinase pathway [12]. Moreover, dandelion polysaccharides may also exert anticancer effects by inhibiting the PI3K/AKT/mTOR pathway and enhancing immune responses [13].

*Melissa officinalis*, or lemon balm, is widely used in traditional medicine and aromatherapy for its calming effects, though it has received less attention for its anticancer potential. Nevertheless, some papers have reported in vitro and in silico studies, phytochemical screening, and also its antioxidant, cytotoxic, and antiproliferative activities, suggesting high potential as a potent therapeutic agent [14,15]. The antiproliferative action of lemon balm has been studied for its chemopreventive effects on lung cancer [16], breast cancer [17], colon cancer [18], and melanogenesis in murine melanocytes [19,20]. Furthermore, aqueous extracts of *M. officinalis* have demonstrated a significant impact on decreasing serum biomarkers of liver damage, showcasing in vivo antioxidant activity and hepatoprotective effects on diethyl nitrosamine-induced hepatocellular carcinoma in rats [21,22,23]. *Clematis vitalba* has been traditionally used for its anti-inflammatory and analgesic properties. Research indicates that extracts from the aerial parts of *Clematis vitalba* exhibit potent anti-inflammatory, antinociceptive, and antipyretic effects, which could be relevant to potential antitumor activity [24,25,26,27,28,29]. Furthermore, polyphenolic compounds from the *Clematis* genus have been reported to possess cytotoxic effects against various cancer cell lines [29]. The selection of *Clematis vitalba* was based on its well-documented traditional medicinal use, particularly for its anti-inflammatory and analgesic properties, which are relevant in the context of cancer progression [24]. Although other *Clematis* species, such as *C. chinensis* and *C. mandshurica*, have been studied for their cytotoxic effects, *Clematis vitalba* remains unexplored in this regard, providing an opportunity to expand the scientific understanding of the genus. Additionally, its rich phytochemical profile—including flavonoids, saponins, and alkaloids—suggests potential biological activity relevant to tumor suppression. Given that chronic inflammation and oxidative stress play a crucial role in cancer development, we hypothesize that *Clematis vitalba*, like other *Clematis* species, may exhibit antiproliferative effects. Our study aims to provide the first insights into its possible role as an anticancer agent, thereby contributing novel findings to the field. The selection was primarily based on its pharmacological relevance.

Those studies, together with their excellent antioxidant, chemopreventive, and antiproliferative effects, highlighted the three plants as untapped resources in the fight against cancer. Moreover, in a previous phytochemical study of Romanian autochthonous flora, extracts of *Taraxacum officinale*, *Melissa officinalis*, and *Clematis vitalba* were between the plants with the highest therapeutic potential, eliciting further study [29].

Research into plant-synthesized nanoparticles or nanoformulations has introduced a new dimension to the study of plant-derived compounds for anticancer therapies [30,31,32]. For example, nanoparticles synthesized from *Melissa officinalis* and dandelion have demonstrated promising antitumor effects [33,34]. These nanoparticles exhibit enhanced bioavailability and targeted delivery, which can significantly improve therapeutic outcomes while minimizing side effects. However, the full potential of these nanoparticles, particularly when directly compared to existing synthetic anticancer agents, warrants further investigation. Additionally, there is currently no evidence of studies exploring the antitumor effects of nanoparticles derived from *Clematis vitalba* or the simultaneous antitumoral effects of synthetic anticancer drugs combined with silver nanoparticles from plants.

The aim of this study is to investigate the synergistic antitumor effects of plant-derived nanoparticles synthesized from *Melissa officinalis* and *Clematis vitalba* in conjunction with conventional hepatic anticancer drugs.

Previous studies have demonstrated the antitumoral activities of dandelion and their biogenic nanoparticles, revealing significant potential of these plants and their nanoparticles on several tumor lines, showing comparative toxicity with cisplatin and doxorubicin, two important chemotherapeutic agents used in the treatment of certain tumors: breast tumors, colorectal cancer, and hepatocellular carcinomas [34]. In this study, the synthesized nanoparticles were examined for their synergistic effects alongside antitumoral medications for the treatment of liver cancer. Sunitinib and imatinib were selected as synthetic anticancer drugs used as a reference in this study because they are potent, new-generation molecules that act as tyrosine kinase inhibitors with substantial antitumor activity in different types of cancer, even in some refractory metastatic carcinomas or advanced unresectable hepatocellular carcinoma [35,36,37,38,39]. Although both have an apparently identical mechanism of action, such anticancer drugs have different activation sites (sunitinib is a pan-kinase inhibitor; imatinib is a BCR-Abl tyrosine kinase inhibitor), which justifies their selection as control drugs in this study precisely as a result of their particular specificity.

The study evaluates their combined impact on the viability, proliferation, and apoptotic pathways in hepatocellular carcinoma cells in comparison to their effects on normal hepatic cells. Through this research, we assess the potential enhancement in therapeutic efficacy and reduction in adverse effects, paving the way for innovative treatment strategies in liver cancer therapy.

## 2. Materials and Methods

### 2.1. Plant Materials and Plant Extracts Preparation

Fresh leaves of *Melissa officinalis* L. were collected from a cultivated garden in Dâmboviţa County in May 2024. Flowers and leaves of *Taraxacum officinale* L. and *Clematis vitalba* L. were harvested from Argeș County in July 2024. The plants were identified and authenticated in the Botanical Laboratory at Titu Maiorescu University, Faculty of Pharmacy, Bucharest.

The plants were dried at room temperature, in a shaded area, for 10 days and subsequently ground using a grinder. Extracts were prepared by refluxing 10 g of the dried plant material with 90 g of a 30% ethanol solution (*w*/*w*) for dandelion and lemon balm (30% ethanolic extracts) and water for *Clematis* sp. (aqueous extract) in a water bath for 30 min. The mixture was then filtered using filter paper and the resulting extracts were stored in a refrigerator at 4 °C [40]. 

### 2.2. Silver Nanoparticle Biosynthesis

For the synthesis of silver nanoparticles (AgNPs), the process began by heating the magnetic plate and placing a beaker containing the 0.5 mM AgNO_3_ (Sigma Aldrich, St. Louis, MO, USA) solution on it. The solution was then allowed to reach 60 °C while maintaining continuous magnetic stirring. The plant extracts were added from the cylinder using a Pasteur pipette at a rate of 45 drops per 30 s. Falcon tubes were then prepared for sampling during the synthesis process. At each observed color change, approximately 2 mL of the sample was taken and placed into the appropriately labelled Falcon tubes for subsequent UV-Vis spectroscopic analysis of the maximum absorption characteristics associated with the formation of silver nanoparticles.

### 2.3. Physical–Chemical Characterization Methods of Nanoparticles

The absorption properties of AgNPs were assessed using a model U-0080D UV-Vis photodiode array spectrophotometer (Hitachi, Tokyo, Japan). The hydrodynamic diameter and surface charge of NP-Ag were analyzed using a Beckman Coulter Delsa Nano C equipped with dynamic light scattering (DLS) and electrophoretic light scattering (ELS) capabilities. 

Time-dependent fluctuations of laser light intensity were generated by illuminating the nanoparticles with a dual 30 mW laser diode. Morphological investigation of the nanoparticles was conducted using a Nova NanoSEM 630, a field emission scanning electron microscope (FE-SEM) from FEI Company, USA, operating at an acceleration voltage of 10.0 kV and a magnification of 2,000,000×.

### 2.4. Cytotoxicity Evaluation of Cell Cultures and Treatments—In Vitro Antitumoral Tests

The cytotoxic potential of the investigated plant extracts, their combination with nanoparticles, with antitumor drugs, and anticancer drugs used as control groups (sunitinib and imatinib) was assessed on a standardized adherent human cancer cell line and compared to their effects on normal human endothelial cells using the oncolytic drug commonly employed in cancer therapy as a benchmark. The human hepatic adenocarcinoma cell line (HepG2, HB-8065) and human umbilical vein endothelial cells (HUVEC, CRL-1730) were obtained from the American Type Culture Collection (ATCC, Manassas, WV, USA).

Sunitinib capsules 50 mg (Sunitinib^®^—Accord, Barcelona, Spain) and Imatinib tablets 100 mg (Imakrebin^®^—Alvogen, Luxembourg) were used as classic anticancer agents to treat the cell control groups. Working solutions were prepared fresh for each experiment through serial dilutions of stock solutions in the culture medium. Adherent cells were maintained in a DMEM/F12 medium supplemented with 10% fetal bovine serum, 2 mM L-glutamine, 100 units/mL penicillin, and 100 μg/mL streptomycin (Sigma Aldrich, St. Louis, MO, USA). The cultures were incubated at 37 °C in a humidified environment with 5% CO. Upon reaching approximately 60% confluence after 24 h, the cells were treated with 100 μL of various concentrations of the test samples for 24 h and 48 h. Following treatment, cells were detached using a nonenzymatic PBS/1 mM EDTA solution, washed twice in PBS, and subsequently utilized in cytotoxicity assays. Untreated cells served as controls throughout the experiments.

- MTS-Based Cytotoxicity Assay

Cell viability was assessed using the MTS-based colorimetric assay, CellTiter 96^®^ AQueous One Solution Cell Proliferation Assay (Promega, Madison, WI, USA). The experiments were conducted in triplicate using 96-well flat-bottom microtiter plates (Falcon, Teterboro, NJ, USA). This assay measures cell viability by quantifying the reduction of MTS, a yellow tetrazolium salt (MTS; Owen’s reagent), into a soluble colored formazan product by metabolically active cells [41]. A total of 1 × 10 cells per well were seeded in 100 μL of culture medium and incubated for 24 h. Following incubation, the culture supernatants were removed and the cells were exposed to increasing concentrations of the test drugs for 24 and 48 h. After the treatment period, 20 μL of the reagent, containing (a) MTS [3-(4,5-dimethylthiazol-2-yl)-5-(3-carboxymethoxyphenyl)-2-(4-sulfophenyl)-2H-tetrazolium, inner salt] and (b) PES (phenazine ethosulfate), was added to each well. The plates were incubated for an additional 4 h at 37 °C with gentle agitation every 15 min. The reduction of MTS to formazan was quantified spectrophotometrically at a wavelength of 492 nm using a DYNEX Technologies MRS plate reader (Dynex, Philadelphia, PA, USA). The percentage of cell viability relative to untreated control cells (considered 100% viable) was calculated using the following formula:

$$\mathrm{Cell}\;\mathrm{Viability}\;\left(\%\right)=\frac{\mathrm{absorbance}\;\mathrm{of}\;\mathrm{treated}\;\mathrm{cells}\;-\mathrm{absorbance}\;\mathrm{of}\;\mathrm{culture}\;\mathrm{medium}}{\mathrm{absorbance}\;\mathrm{of}\;\mathrm{untreated}\;\mathrm{cells}\;-\mathrm{absorbance}\;\mathrm{of}\;\mathrm{culture}\;\mathrm{medium}}\times 100$$

The percentage of cell viability relative to untreated control cells was determined and the results were reported as the mean ± standard deviation (SD) from experiments conducted in triplicate (*n* = 3).

### 2.5. Preparation of In-Vitro-Tested Samples

For each of the three plant extracts studied (*Taraxaci extractum*—TE, *Melissae extractum*—ME, *Clematis vitalbae extractum*—CVE) prepared according to the procedure described in Section 2.1., successive serial dilutions of 1:1, 1:2, 1:4, 1:8, 1:16, 1:32, 1:64, and 1:128 in deionized water were carried out. Subsequently, the samples thus processed were subjected to the MTS cytotoxicity assay.

Also, samples with silver nanoparticles obtained according to the biosynthesis described in Section 2.2. were prepared in serial dilutions from 1:1, 1:2, 1:4, 1:8, 1:16, 1:32, 1:64 to 1:128 with deionized water.

Two control groups were used for the reference antitumor action, consisting of Sunitinib (SNTB) and imatinib (IMTB). The control samples were prepared following the same preparation conditions for successive dilutions from 1:1 to 1:128 as those for test samples. Compared to the anticancer action of these two synthetic drugs used in clinical practice, all the cytotoxic results obtained for each plant extract, plant extract–nanoparticle combination, or plant extract ± nanoparticle–drug combination were reported and compared.

All the abbreviations used in this study to define the treatments applied to HEPG2 and HUVEC cells are presented and explained below in Table 1 for *Taraxaci extractum* treatment groups, in Table 2 for *Melissae extractum* treatment groups, in Table 3 for *Clematis vitalbae extractum* treatment groups, and in Table 4 for control treatment groups.

### 2.6. Statistical Analysis

Statistical analysis was carried out using IBM SPSS Statistics Software Version 29.0.2.0 (20) (IBM Corporation, Chicago, IL, USA) in order to evaluate the statistically significant differences of the tested groups (TE-derived samples, ME-derived samples, and CVE-derived samples) between the control ones (SNTB and IMTB). The essential assumptions for the application of statistical tests (such as the continuity of variables, the normality and linearity of data, the absence of outliers, the homogeneity of variances, and the independence of observations) were evaluated for each set of experimental data before running the parametric statistical assays. Normality tests (Kolmogorov–Smirnov test and Shapiro–Wilk test), Levene’s test for the homogeneity of variances, and robust tests of equality of means (Welch *t*-test and Brown–Forsythe’s test) were implemented to assess and verify data analysis criteria [42,43,44,45]. When certain experimental datasets deviate from the normal distribution criteria or some mandatory assumptions are violated, a two-step transformation procedure is used for resistant datasets so that they become normally distributed and can be subjected to one-way analysis of variance (ANOVA), followed by post hoc tests. We chose to apply a modified version of the Games–Howell post hoc test instead of the Dunnett test, in which we were only interested in the differences between each treatment group and the control group because we have unequal size groups with unequal variances (α < 0.05). All the results were expressed as means and standard deviations (mean ± SD) for at least three replications for each sample (*n* = 3). The chosen level of significance was set at 0.05 and the results were considered statistically significant when *p* < 0.05.

### 2.7. IC 50, Selectivity Index

IC (inhibitory concentration) curves are dose-response curves used to determine the specific concentration of a drug required to reduce the population of viable cells by a certain percentage, compared to cells grown without exposure to the drug. They illustrate a population change of increased cell death or decreased cell proliferation. Determination of the half-maximal (50%) inhibitory concentration (IC50) is essential for understanding the pharmacological and biological characteristics of a treatment and is presented as mean ± error, with the error being given by the four-parameter logistic regression (4PL) to the data. The regression was implemented in Python using the scipy curve_fit function:

The selectivity index (SI) is a crucial metric in drug discovery and cancer research. It measures how selectively a compound kills cancer cells compared to normal cells. It is calculated using the following Formula (1):

(1) $$\mathrm{SI}\;=\frac{\mathrm{IC}50\;\left(\mathrm{normal}\;\mathrm{cells}\;\mathrm{HUVEC}\right)}{\mathrm{IC}50\;\left(\mathrm{cancer}\;\mathrm{cells}\;-\;\mathrm{HepG}2\right)}$$

### 2.8. Chou–Talalay Method

To determine the combination index (CI), the viability data have been converted to inhibition data and a Hill function has been fitted to the thus obtained data, resulting in the parameters necessary for its calculation using the Chou–Talalay Method [46,47]:

(2) $$\frac{I}{I_{\max}}=\frac{{\left[A\right]}^{m}}{{\mathrm{IC}}_{50}^{m}+{\left[A\right]}^{m}}$$

where [A] is the compound concentration, I is the measured inhibition for the given concentration, and m is the coefficient determining the shape of dose-effect curve (m < 1, m = 1, and m > 1, correspond to negative sigmoidal, hyperbolic, and sigmoidal curves, respectively). Moreover, for each fit, a conformity value is calculated, represented by the linear correlation coefficient, r. In the Chou–Talalay equations, IC50, the dose of 50% effect is substituted by Dm. For two drug mixtures, as employed in this article, the CI is calculated using:

(3) $$\mathrm{CI}=\frac{{\left(D\right)}_{1}}{{\left(D_{x}\right)}_{1}}+\frac{{\left(D\right)}_{2}}{{\left(D_{x}\right)}_{2}}$$

where (D) represents the dose of the compound and (D_x_) represents the dose of the compound that exert x% inhibition in combination.

Moreover, given that the doses of the two compounds sum to the (D_x_) of the combination and that the (D_x_) for any compound can be deduced from the median effect equation:

(4) $$\left(D_{x}\right)=\mathrm{Dm}\;{\left(\frac{\mathrm{fa}}{1-\mathrm{fa}}\right)}^{1/m}$$

Equation (2) can be rearranged into:

(5) CI=(Dx)1,2 D1D1+D2Dm1fa1−fa1/m1+(Dx)1,2 D2D1+D2Dm2fa1−fa1/m2

where fa represents the fraction affected by the drug dose for which the combination index is calculated. The calculation of the CI has been performed using a Python 3.12 algorithm, with the fitting parameters being obtained using the curve_fit function from the scipy library. Additivity was considered when the CI was close to 1 [0.9–1.1], antagonism when CI > 1, and synergy when CI < 1.

## 3. Results and Discussions

### 3.1. Physical–Chemical Nanoparticles Characterization

- UV-VIS Spectrometry

**Figure 1:**
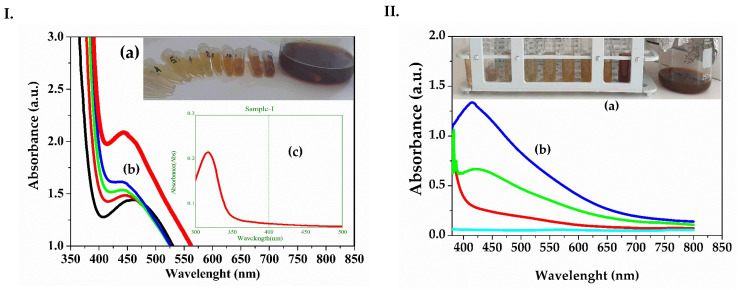
(**I**). (a) Color change in the solution from brownish-red to a dark brown due to silver bioreduction by *Melissa officinalis* extract; (b) UV–Vis spectra of ME AgNPs samples obtained by reduction in time with *Melissa officinalis* extract; (c) UV–Vis spectra of 0.5 mM AgNO_3_ aqueous solution; (**II**). (a) Color change in the solution from brownish-red to a dark brown due to silver bioreduction by *Clematis vitalba* extract; (b) UV–Vis spectra of CVE AgNPs samples obtained by reduction in time with *Clematis vitalba* extract. ME AgNPs: *Melissae extractum* silver nanoparticles; CVE AgNPs: *Clematis vitalbae extractum* silver nanoparticles.

Figure 1I and II show the UV-Vis spectra for the recorded samples during the synthesis of silver nanoparticles (AgNPs) from *Melissa officinalis* (ME AgNPs) and *Clematis vitalba* (CVE AgNPs), respectively. The methods for obtaining the dandelion extract were previously presented in our study, with the maximum absorbance for dandelion silver nanoparticles synthesized at 462 nm from the ethanolic extract and 450 nm for AgNPs from the aqueous extract [34]. As seen in Figure 1(Ia,IIa), there was a change in color from a brownish-red to a dark brown after mixing the AgNO_3_ solution with the extracts. This color change was attributed to the formation of silver nanoparticles. Additionally, the formation of the nanoparticles was confirmed by the appearance of absorption maxima with values between 400–460 nm for both syntheses. Figure 1(Ib), regarding the formation of ME AgNPs, showed an initial absorption maximum of 453 nm, which then shifted to a lower value and stabilized at 440 nm after 30 min for ME AgNPs. The volume ratio between the 0.5 mM AgNO_3_ solution and the *Melissa* extract at the end of the synthesis was 3.2:1 (v:v). In the case of the formation of silver nanoparticles from *Clematis vitalba*, the absorption maximum eventually stabilized at a lower value of 413 nm. The lower values of the absorption maximum recorded for silver nanoparticles were correlated in the literature with smaller nanoparticle sizes [48]. Thus, for the silver nanoparticles from *Clematis vitalba*, smaller nanoparticle sizes were obtained compared to those from *Melissa*, though the volume added from the extract was slightly greater, with the ratio between the Ag salt and the *Clematis* extract being 2:1 (v:v).

- Dynamic Light Scattering (DLS), Zeta Potential, and Scanning Electron Microscopy (SEM)

In this study, dynamic light scattering (DLS), electrophoretic light scattering (ELS), and scanning electron microscopy (SEM) were employed to analyze the interactions of two types of biogenic silver nanoparticles in their colloidal final forms post-biosynthesis. Although the plant extracts were filtered prior to being used for nanoparticle biosynthesis, the nanoparticles themselves were not purified, leading to the possibility of larger extract particles remaining alongside the nanoparticles.

**Figure 2:**
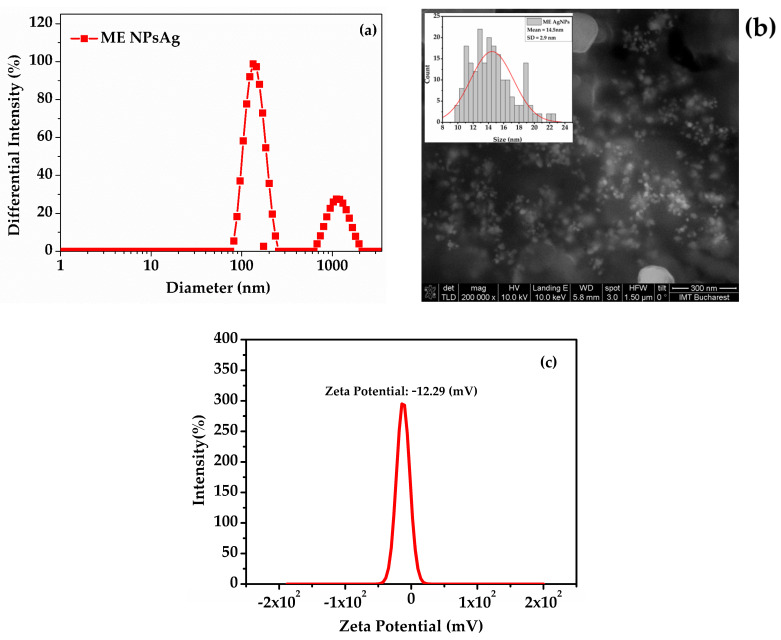
(**a**) DLS measurements of ME AgNPs; (**b**) SEM (Scanning Electron Microscopy) images of ME AgNPs; (**c**) Zeta potential of ME AgNPs, with a value of −12.29 mV. ME AgNPs: *Melissae extractum* silver nanoparticles.

Figure 2a illustrates the intensity-based size distributions of silver nanoparticles synthesized using *Melissa officinalis* extracts (ME AgNPs). The size distribution profile revealed two populations of particles: a broad distribution ranging from 75 nm to 255 nm centered at 143 nm and a larger-sized population centered at 1108 nm; the latter attributed to larger particles from the *Melissa officinalis* extract. The SEM image of the colloidal solution with the nanoparticles and *Melissa* extract (Figure 2b), after dehydration, indicated that the nanoparticles were considerably smaller than those determined by DLS, suggesting that they were coated with extract negative charge compounds. This observation is corroborated by the stable negative zeta potential of −12.2 mV for the *Melissa* extract nanoparticles, as shown in Figure 2c.

For the nanoparticles synthesized from *Clematis vitalba*, their very small size led to aggregation, confirmed by an increase in hydrodynamic diameter and, consequently, resulted in a negative but very low zeta potential, indicating instability in the resultant nanoparticle solution. Figure S1a–c in the Supplementary Materials presents the hydrodynamic diameter, SEM image with inset hystogram, and the recorded zeta potential for the silver nanoparticles derived from *Clematis vitalba.* The nanoparticle diameter of the CVE AgNPs and ME AgNPs is presented in Table 5.

The size distribution of the CVE AgNPs and ME AgNPs samples was determined from SEM images by measuring approximately 200 nanoparticles per sample. The open-access program ImageJ (version 1.53t) was utilized to extract the necessary data from the SEM micrographs and generate the corresponding histograms.

The nanoparticle sizes ranged from 6 nm to 18 nm for CVE AgNPs and 9 nm to 22 nm for ME AgNPs. These measurements were compiled into histograms, which were accurately modeled using Gaussian fitting to represent the distribution.

The full width at half maximum (FWHM) analysis revealed that the highest percentage of nanoparticles for CVE AgNPs fell within the size range of 9–13 nm (as seen in Figure S1), while for ME AgNPs, it was within 11–17 nm (inset of Figure 2b). The mean diameter of the nanoparticles was calculated to be 11.1 ± 1.9 nm for CVE AgNPs and 14.5 ± 2.9 nm for ME AgNPs, highlighting a slightly larger size distribution for the ME AgNPs sample. These findings provide a detailed characterization of the nanoparticle size distribution, essential for understanding their morphological and functional properties.

Furthermore, the stability of silver nanoparticles was evaluated using dynamic light scattering (DLS) at 12 h, 48 h, and 72 h after synthesis as well as one month post-synthesis with nanoparticle solutions being stored at room temperature in between measurements. The three samples were analyzed in terms of hydrodynamic diameter, zeta potential, and polydispersity index, with the results being presented in Table 6. The evolution of the hydrodynamic size highlights significant differences between the synthesis methods. ME AgNPs 3% ETOH shows a progressive size reduction over time, indicating particle maturation and possible stabilization through a ripening process. Meanwhile, CVE AgNPs aqueous exhibits a pronounced decrease in size after 48 h, suggesting particle aggregation and partial destabilization. Moreover, the polydispersity index (PDI) trends reveal differences in colloidal stability. TE AgNPs 5% ETOH maintains a relatively stable PDI over time, indicating a homogeneous particle size distribution. Conversely, aqueous CVE AgNPs start with a very high PDI (1.588) at 24 h, pointing to significant size heterogeneity, which gradually reduces, reflecting partial aggregation or sedimentation. Zeta potential measurements also indicate variations in colloidal stability. TE AgNPs 5% ETOH consistently maintains a zeta potential below −30 mV, indicating good electrostatic stability, whereas ME AgNPs 3% ETOH shows a decline in zeta potential from −31.04 mV to −12.29 mV over one month, suggesting reduced stability and possible particle aggregation. CVE AgNPs aqueous exhibits fluctuations, with a shift from −34.05 mV to −27.54 at 72 h, followed by a drop to −0.60 mV after one month, signaling severe destabilization. These results suggest that the solvent may play an important role in nanoparticle stability, as the addition of a small amount of ethanol could contribute to the stabilization of ME and TE AgNP solutions, while the aqueous extract in CVE AgNPs may have led to poorer long-term colloidal stability. However, this hypothesis requires further investigation to fully confirm the influence of the solvent on stability. Comparative analysis of synthesis methods shows that TE AgNPs 5% ETOH offers the highest long-term colloidal stability, as indicated by relatively constant particle size, low PDI, and stable zeta potential. In contrast, CVE AgNPs aqueous demonstrates the least stability, with large size fluctuations, high polydispersity, and unstable zeta potential, suggesting limited suitability for long-term applications without additional stabilizing agents.

The ability of these plants to produce a negative zeta potential on nanoparticles is closely linked to their acidic functional groups (e.g., carboxyl and hydroxyl groups from polyphenols and flavonoids). Among the three plants, dandelion and lemon balm are more likely to generate a stronger negative zeta potential due to their higher polyphenolic and flavonoid content, which can impart a stable, negatively charged surface on the nanoparticles [49]. Meanwhile, *Clematis vitalba* may have a weaker or less consistent negative zeta potential due to its lower or less diverse content of such stabilizing compounds, though further quantitative zeta potential measurements are required for confirmation. All three plant extracts have the potential to produce a negative zeta potential; dandelion and lemon balm are expected to yield stronger, more stable negative charges due to their richer composition of polyphenols and flavonoids, whereas *Clematis vitalba* may require further investigation to confirm its stabilizing capacity in nanoparticle suspensions. The significant discrepancy between the hydrodynamic diameter measured by DLS and the particle size observed via SEM may be attributed to the presence of organic capping agents from extracts (especially for CVE), which form a hydration layer around the nanoparticles, increasing their apparent size in solution. Additionally, DLS is highly sensitive to nanoparticle aggregation, which can lead to an overestimation of size, especially in polydisperse samples with high PDI values. Similar observations regarding the effect of capping agents and aggregation on DLS measurements have been reported in previous studies [50,51].

### 3.2. In Vitro Antitumoral Studies

- Taraxaci extractum (TE)-based samples

**Figure 3:**
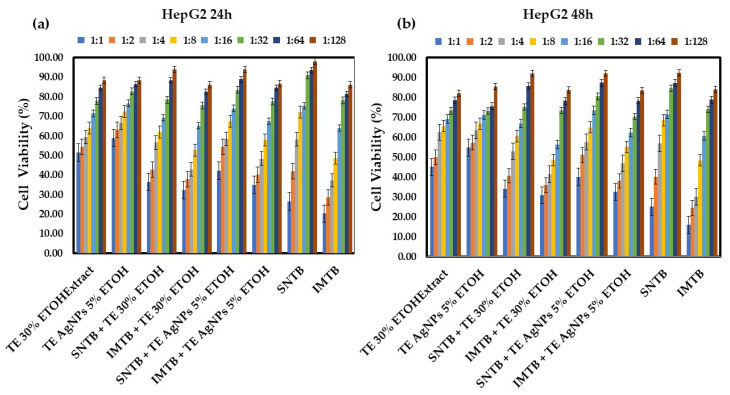
Cell viability (%) of HepG2 lines after: (**a**) 24 h of treatment with various dilutions of *Taraxaci extractum*-based samples and the chemotherapeutic drugs sunitinib and imatinib; (**b**) 48 h of treatment with various dilutions of *Taraxaci extractum*-based samples and the chemotherapeutic drugs sunitinib and imatinib. Error bars represent standard deviation (*n* = 3). The abbreviations are explained in Table 1 and Table 4. Different colors represent various dilution ratios (v:v) for each sample with deionized water.

Figure 3a,b illustrate the cell viability in the treatment of the HepG2 liver tumor cell line with samples derived from dandelion after 24 h and 48 h. Different concentrations of treatments were applied to the HepG2 liver tumor cell line. Variation in values suggests differing treatment effectiveness based on concentration and time. Normality histograms and normal Q-Q plots for each TE group versus each control group on HepG2 cells are presented in Supplementary Materials Figures S2–S5.

At 24 h, significant inhibition of cell viability was observed at dilutions 1:1, 1:2, and 1:4, with a noticeable antitumor effect. The combination of the 30% ethanol extract of *Taraxacum* exhibited nearly the same cytotoxic effect as its combination with silver nanoparticles. However, the addition of nanoparticles did not significantly enhance the antitumor effect on HepG2 cells at this time point.

After 48 h, the treatments exhibited similar trends, with lower dilutions (1:8 and 1:16) becoming more effective over time. Nonetheless, the samples containing silver nanoparticles did not show any statistically significant improvement (*p* > 0.05) in antitumor efficacy compared to conventional chemotherapeutic agents. The toxicity of *Taraxacum*-derived samples on normal HUVEC cells was also investigated. While a cytotoxic effect on hepatic tumor cells was observed at both 24 and 48 h, it is preferable that these treatments do not exert toxic effects on normal endothelial cells. However, a slight improvement in cell viability was noted when sunitinib was administered alongside silver nanoparticles, suggesting that silver nanoparticles might mitigate the toxicity of sunitinib on endothelial cells.

Although the results obtained for dandelion-based samples, compared to the control groups (sunitinib and imatinib), were not statistically significant, it can be observed that the viability of the liver tumor cells of the groups treated with TE AgNPs and synthetic drugs is very close to those obtained for the control groups.

Furthermore, the toxic effects of dandelion-derived samples on the normal HUVEC cell line were investigated. While a cytotoxic effect on hepatic tumor cells was clearly observed at both 24 and 48 h, it is preferable that these samples do not exert toxic effects on normal HUVEC cells. The cytotoxic effect of dandelion-derived samples was also observed in HUVEC cells, similarly to chemotherapeutic drugs. However, a slight improvement in cell viability was noted when sunitinib was administered alongside silver nanoparticles, indicating that the toxicity of sunitinib on endothelial cells is mitigated by the addition of silver nanoparticles.

Silver nanoparticles biosynthesized from dandelion have been documented in several studies to possess antioxidant effects, thereby potentially protecting cells from oxidative stress induced by sunitinib [9]. The viability of HUVEC cells after 24- and 48-h post-treatment with dandelion-based samples is presented in the Supplementary Materials Figure S6.

- Melissae extractum (ME)-based samples

Studies indicate that the ethanolic extract of *Melissa officinalis* exhibits cytotoxic effects on colon and breast cancer cells, primarily through the induction of apoptosis and the generation of reactive oxygen species [4,5,17,18]. Additionally, its active compounds, particularly rosmarinic acid, contribute to antiproliferative and antimigratory effects, suggesting potential for chemoprevention in breast cancer and also in hepatocellular carcinoma [6,21]. Furthermore, the cytotoxic effect of this plant has also been demonstrated on the HepG2 cellular line, an effect that was concentration dependent [23]. 

**Figure 4:**
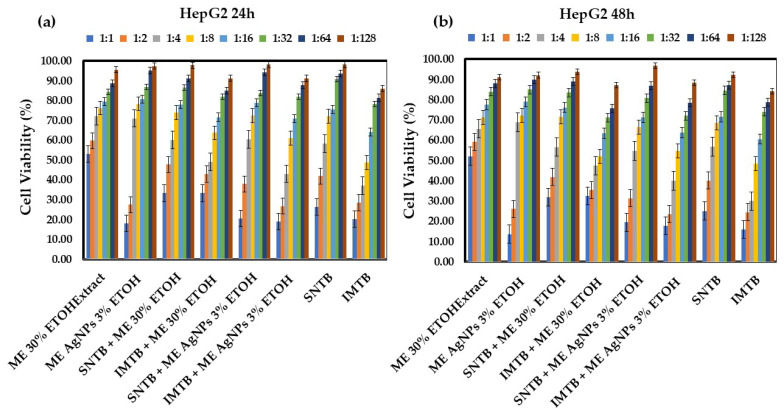
Cell viability (%) of HepG2 lines after: (**a**) 24 h of treatment with various dilutions of *Melissae extractum*-based samples and the chemotherapeutic drugs sunitinib and imatinib; (**b**) 48 h of treatment with various dilutions of *Melissae extractum*-based samples and the chemotherapeutic drugs sunitinib and imatinib. Error bars represent standard deviation (*n* = 3). The abbreviations are explained in Table 2 and Table 4. Different colors represent various dilution ratios (v:v) for each sample with deionized water.

The treatments outlined in this study aim to utilize silver nanoparticles derived from *Melissa officinalis*, which have also been shown to play a significant role in inhibiting tumor cell proliferation, exhibiting a more potent effect than the extract alone. The combination of the alcoholic extract and biogenic silver nanoparticles obtained from *Melissa officinalis* (ME AgNPs) may enhance treatment efficacy and could be as effective, or even more effective, with minimal toxic effects on normal cells. Thus, the cytotoxic effect of the extracts derived from *Melissa officinalis* was evaluated after 24 h and 48 h post-treatment. Figure 4a,b present measurements of cell viability after 24 h and, respectively, 48 h of treatment with the extracts derived from *Melissa officinalis* compared to the drugs administered alone.

At 48 h, the antitumor effect is enhanced for the dilutions of silver nanoparticles from *M. officinalis* at ratios of 1:1 and 1:2 (ME AgNPs 1:1 and 1:2). Combination therapy with these medications potentiates their antitumor effect compared to the administration of the drugs alone.

In the case of *M. officinalis* nanoparticles added following treatment with medications, they demonstrated remarkable efficacy at the first and second dilutions, proving to be even more potent than the chemotherapeutic agents sunitinib and imatinib.

In Table 7 and Table 8, the statistically significant results obtained compared to the control group can be identified (SNTB + ME AgNPs 1:1 vs. SNTB (*p* = 0.002) and IMTB + ME AgNPs 1:1 vs. IMTB (*p* = 0.036)). So, the established antitumor effect of *M. officinalis* is enhanced when it is formulated as a colloidal solution of silver nanoparticles resulting from biosynthesis. Furthermore, other authors have observed in their studies the effectiveness of silver nanoparticles derived from *M. officinalis* on the HepG2 cell line, which induces apoptosis. According to their findings, the activation of apoptosis may be attributed to the excessive production of reactive oxygen species (ROS), thereby affecting the tumor cell DNA [52].

Among the two drugs, sunitinib exhibits a weaker antitumor effect (25.73% HepG2 cell viability) compared to imatinib (18.08% HepG2 cell viability). When used in combination with nanoparticles, the antitumor efficacy surpasses that of the drugs administered alone (SNTB + ME AgNPs 1:1 vs. SNTB: 20.01% vs. 25.73% (*p* = 0.002) and IMTB + ME AgNPs 1:1 vs. IMTB: 17.80% vs. 18.08% (*p* = 0.036)).

A slight increase in the proliferation of normal cells is observed compared to the administration of the drugs alone at low dilutions when combinations with nanoparticles are employed. This also confirms findings related to dandelion, where the combination with silver nanoparticles reduces the toxicity of the medications over normal cells, HUVEC.

Normality histograms and normal Q-Q plots for each ME group versus each control group on HepG2 cells are presented in Supplementary Materials Figures S7–S10.

Statistically significant differences compared to the control group (SNTB or IMTB) were detected for ME AgNPs administered alone: ME AgNPs 1:1 vs. SNTB (*p* = 0.003), ME AgNPs 1:2 vs. SNTB (*p* = 0.022), and ME AgNPs 1:1 vs. IMTB (*p* = 0.028). However, the nanoparticles alone (ME AgNPs) exhibit the highest toxicity at elevated concentrations, becoming toxic to normal HUVEC cells as well. The toxicity toward normal cells manifests at high concentrations and when only extracts combined with the medications are administered. The cytotoxicity of the combination of drugs and nanoparticles on tumor cells is significantly greater than that of the drugs alone at all lower dilutions (1:4), while the toxicity toward normal cells decreases with dilution.

One possible reason for the potent inhibitory effects of *M. officinalis* on HepG2 cells could be that AgNPs reduce adenosine triphosphate (ATP) content in the cells by damaging mitochondria, which, in turn, increases the production of reactive oxygen species (ROS) in a dose-dependent manner. The anticancer effect of silver nanoparticles might also be attributed to their small size (<15 nm) and the doping of secondary metabolites, such as phenols or flavonoids, on their surface, leading to mitochondrial damage, increased l production, and apoptosis.

The cell viability (%) of the HUVEC cell line measured for samples derived from *M. officinalis* is presented in the Supplementary Materials Figure S11.

- Clematis *vitalbae extractum* (CVE)-based samples

**Figure 5:**
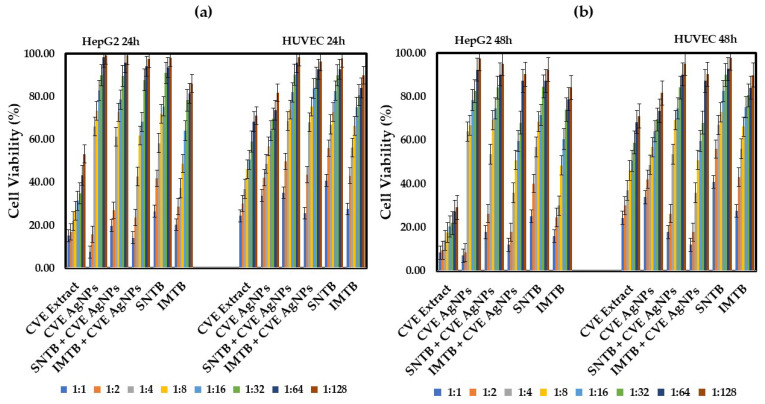
Cell viability (%) after: (**a**) 24 h of treatment of HepG2 and HUVEC by *Clematis vitalbae extractum*-based samples; (**b**) 48 h of treatment of HepG2 and HUVEC by *Clematis vitalbae extractum*-based samples. Error bars represent standard deviation (*n* = 3). The abbreviations are explained in Table 3 and Table 4. Different colors represent various dilution ratios (v:v) for each sample with deionized water.

Figure 5a,b illustrate the cellular viability following treatment of HepG2 cells with samples derived from the species *Clematis vitalba*. Numerous studies on *Clematis* species have identified constituents such as flavonoids, triterpenoid saponins, lignans, steroids, polyphenols, and coumarins [24,53,54]. Notably, several compounds, particularly flavonoids and alkaloids, exhibit substantial evidence of biological significance [55]. Among the three extracts analyzed, *Clematis vitalba* demonstrated the most pronounced cytotoxicity on the HepG2 hepatic cell line. This cytotoxic effect persisted for 24 h and for 48 h even at the greatest dilution of 1:128. Furthermore, when combined with silver nanoparticles (CVE AgNPs), the cytotoxicity was reduced but still remained statistically significant (*p* < 0.05) at the initial two dilutions (1:1 and 1:2).

It is important to highlight that the cytotoxicity of the *C. vitalba* extract on HUVEC cells was observed at a lower magnitude. However, normal cellular proliferation was unaffected at higher dilutions—dilutions at which the extract effectively inhibited the proliferation of hepatic tumor cells.

An additional noteworthy finding was that both the extract and the derived samples, including nanoparticles and their combination with drugs, exhibited a more potent antitumor effect compared to the administration of synthetic drugs alone, especially at dilutions up to 1:2 or 1:4 as seen in Table 9 and Table 10. Furthermore, the proliferation of the normal HUVEC cell line was not as significantly impacted by these treatments as it was by the conventional drugs used for liver cancer treatment, up to the aforementioned dilution level. 

All applied treatment groups were compared separately on the two control groups (sunitinib—SNTB and imatinib—IMTB) and, therefore, the statistical analysis confirms our observations because statistical significance (*p* < 0.05) was achieved during post hoc tests as follows: CVE 1:1 vs. SNTB (*p* = 0.004), CVE 1:2 vs. SNTB (*p* = 0.010), CVE 1:4 vs. SNTB (*p* = 0.035), CVE AgNPs 1:1 vs. SNTB (*p* = 0.005), CVE AgNPs 1:2 vs. SNTB (*p* = 0.005), SNTB + CVE AgNPs 1:1 vs. SNTB (*p* = 0.000), and SNTB + CVE AgNPs 1:2 vs. SNTB (*p* = 0.018). These concrete results highlight the fact that both CVE (1:1, 1:2, 1:4) and CVE AgNPs (dilution 1:1 and 1:2) determined a significant decrease in the viability of HepG2 tumor cells compared to the synthetic chemotherapeutic drug sunitinib. Between the CVE extract and the CVE extract combined with silver nanoparticles, CVE AgNPs 1:1 showed a more significant cytotoxic effect, inducing a decrease in tumor cell viability of approximately 7.25% compared to CVE 1:1, where the decrease in viability was 11.71%. Thus, the antitumor effect was much more potent than that observed in the control group treated only with sunitinib in the highest 1:1 concentration, where cell viability was 25.73%. In addition, the cytotoxic activity of CVE amplifies as the exposure time of HepG2 cells increases from 24 h to 48 h, especially for the 1:1, 1:2, and 1:4 dilutions, regardless of whether it is the CVE extract, the combination with nanoparticles, or the association with the reference drug. Normality histograms and normal Q-Q plots for each CVE group versus each control group on HepG2 cells are presented in Supplementary Materials Figures S12–S15.

Similar significant results were also highlighted after the comparison with the control group treated only with imatinib, as follows: CVE 1:1 vs. IMTB (*p* = 0.023), CVE AgNPs 1:1 vs. IMTB (*p* = 0.011), CVE AgNPs 1:2 vs. IMTB (*p* = 0.028), and IMTB + CVE AgNPs 1:1 vs. IMTB (*p* = 0.001). The CVE 1:2 influence on hepatic tumor cell viability compared to the IMTB control we considered to be on the edge of significance, although the *p*-value exceeds the established significance threshold (*p* = 0.066 > 0.05) because it is known that the statistical significance is higher when the *p*-value is closer to the alpha value, so this very narrow approach may show that *Clematis vitalbae extractum* 1:2 can bring statistically significant therapeutic benefits in medical research on tumor cells.

It can be noted that the association of CVE AgNPs nanoparticles diluted 1:1 and 1:2 together with classic antitumor drugs like sunitinib or imatinib causes a statistically significant decrease in the viability of HepG2 cells, much lower compared to that evaluated in the control group treated only with the synthetic drug (SNTB + CVE AgNPs 1:1 vs. SNTB: 18.73% vs. 25.73% (*p* = 0.000), SNTB + CVE AgNPs 1:2 vs. SNTB: 26.62% vs. 41.00% (*p* = 0.018), IMTB + CVE AgNPs 1:1 vs. IMTB: 12.99% vs. 18.08% (*p* = 0.001)).

Therefore, it can be stated that the addition of CVE AgNPs 1:1 and 1:2 to the reference antitumor drug causes a synergistic effect, resulting in an increase in the anticancer efficacy of the synthetic drug with remarkable therapeutic benefits in the fight against cancer.

**Figure 6:**
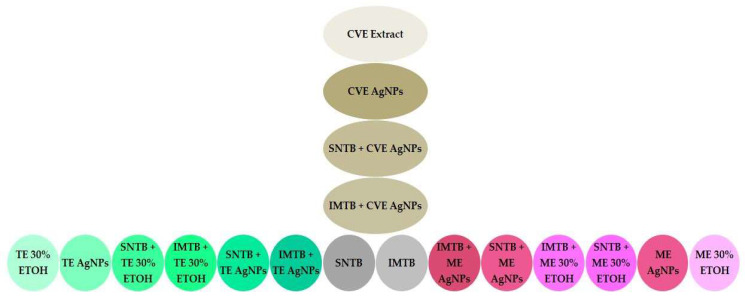
Shaped matrix diagram to compare treatment groups using the intensity of the antitumor effect as a criterion. The abbreviations are explained in Table 1, Table 2, Table 3 and Table 4.

The shaped matrix diagram (Figure 6) was designed to illustrate and compare the antitumor effect for all treatment groups. Using a well-defined color-coding system to separate the different types of treatment makes it easier to observe the difference between the effects caused by each one, find differences in how strong the effects are, and put the studied lots in their correct groups [56,57]. This system allows linking between particular colors and the intensity of effect. Thus, pale and light colors indicate an effect of a lower intensity (lower antitumor effect) and more saturation shows a higher intensity of the anticancer effect for the applied treatments.

### 3.3. IC50 Determination and SI After 24 h and 48 h

- IC50

Table 11 presents the IC50 values of the tested compounds, highlighting the potential of AgNP-based treatments and underscoring the importance of evaluating both selectivity and cytotoxicity for effective anticancer therapy. By comparing IC50 values obtained at 24 h and 48 h for the HUVEC and HepG2 cell lines, significant differences between simple extracts and AgNP-modified counterparts were observed.

Notably, compounds incorporating AgNPs exhibited considerably lower IC50 values, indicating enhanced cytotoxicity on HepG2 cancer cells. For instance, TE AgNPs 5% ETOH demonstrated a sharp reduction in IC50 (1.91 ± 0.30 µg/mL at 24 h and 1.11 ± 2.42 µg/mL at 48 h) compared to the simple TE 30% ETOH extract (5874.16 ± 593.80 µg/mL at 24 h and 30,530.13 ± 36,511.17 µg/mL at 48 h). This result suggests that AgNP functionalization significantly enhances the cytotoxic efficacy of the extract.

Similarly, IMTB and its AgNP-based combinations exhibited lower IC50 values on HepG2 cells, indicating promising antitumor effects. Specifically, IMTB + TE AgNPs 5% ETOH achieved IC50 values of 22.46 ± 1.73 µg/mL at 24 h and 22.68 ± 4.13 µg/mL at 48 h, demonstrating higher efficacy than the non-AgNP variant. These findings support the hypothesis that AgNPs potentiate the biological effects of natural compounds, likely by enhancing cellular uptake and inducing oxidative stress.

- Selectivity Index and Therapeutic Potential

**Figure 7:**
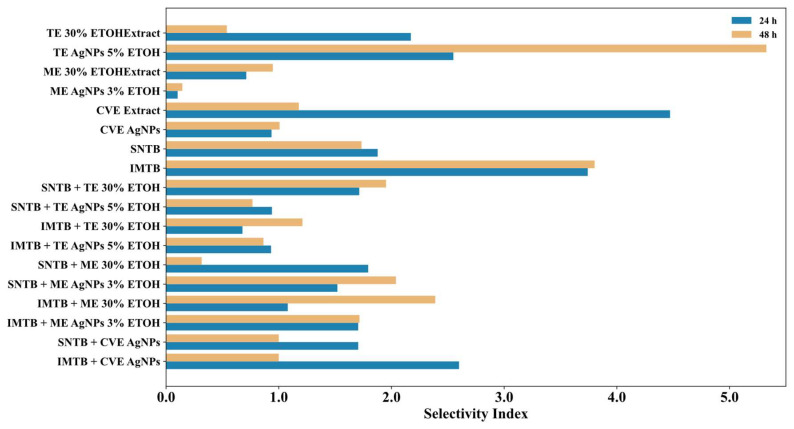
The SI variation of the compounds at 24 h and 48 h; interpretation of SI values: SI > 2: good selectivity (the compound is at least twice as toxic to cancer cells compared to normal cells); SI between 1 and 2: moderate selectivity; SI < 1: poor selectivity (the compound affects both cancer and normal cells similarly).

Figure 7 illustrates the SI for all compounds at 24 h and 48 h, providing a clear perspective on their efficacy on cancer cells relative to their toxicity on healthy cells. Compounds with an SI > 2 are considered selectively cytotoxic toward cancer cells, making them promising candidates for therapeutic applications. The most promising results include: the CVE extract (SI = 4.47 at 24 h) and IMTB (SI = 3.74 at 24 h, 3.80 at 48 h), which demonstrated high cytotoxicity toward HepG2 cells with relatively low toxicity to HUVEC cells. Also, TE AgNPs 5% ETOH showed a marked increase in selectivity (SI = 2.55 → 5.33), indicating consistent therapeutic potential. IMTB + ME 30% ETOH and SNTB + ME AgNPs 3% ETOH maintained SI > 2 at 48 h, suggesting sustained long-term efficacy. Conversely, certain compounds lost selectivity over time including the CVE extract, for which SI decreased from 4.47 at 24 h to 1.18 at 48 h, the IMTB + CVE AgNPs compound, for which SI dropped from 2.60 to 1.00, and the TE 30% ETOH extract, for which SI decreased significantly from 2.17 to 0.54.

These results indicate that some extracts may exhibit time-dependent effects, highlighting the need for formulation optimization to sustain their selectivity.

The IC50 and SI assessments clearly demonstrate that AgNPs combination compounds significantly enhance cytotoxicity and selectivity to HepG2 cancer cells. Among all compounds, TE AgNPs 5% ETOH and IMTB emerged as the most promising candidates, combining potent cytotoxicity with sustained selectivity over time.

These findings emphasize the potential of AgNP-based formulations for anticancer therapies and underscore the importance of further investigations into their molecular mechanisms and long-term effects. Future studies, including in vivo assessments, are essential to confirm their therapeutic efficacy and safety for clinical applications.

- Chou–Talalay Method

The combination index (CI) is a quantitative measure used to evaluate the interactions between two or more drugs when used in combination. The Chou–Talalay method, which is based on the median-effect principle, is commonly used to calculate CI values and determine if drugs act synergistically, additively, or antagonistically.

**Figure 8:**
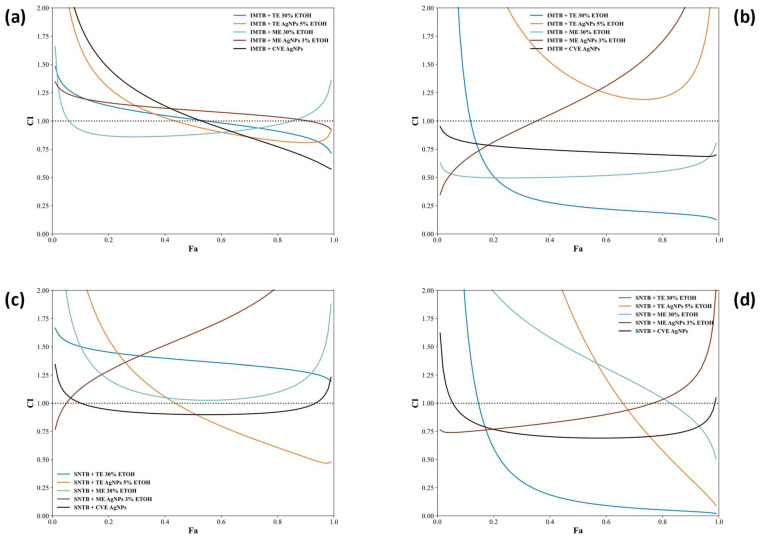
The relationship between the CI and Fa for: (**a**) IMTB combinations compounds at 24 h; (**b**) IMTB combinations compounds at 48 h; (**c**) SNTB combination compounds at 24 h; and (**d**) SNTB combination compounds at 48 h.

Table S1 presents dose-effect parameters (Dm, m, r) and combination index (CI) values for different drug combinations at 24 h and 48 h. The graphs from Figure 8a–d represent the relationship between the CI and the affected fraction of the cells (Fa), which allows the characterization of synergy across all levels of compound potency and dilutions.

The CI values at IC10, IC50, and IC90 provide further insights into the synergistic or antagonistic interactions between drug combinations. CVE-NP + SNTB emerges as the most consistently synergistic pair, with CI values of 0.87 (IC10), 0.69 (IC50), and 0.76 (IC90) indicating strong synergy across all inhibition levels, which could significantly enhance therapeutic efficacy. Similarly, CVE-NP + IMTB maintains synergy throughout (IC10: 0.81, IC50: 0.73, IC90: 0.69), highlighting its potential as a powerful anticancer combination. On the other hand, TE-NP + SNTB displays a dose-dependent effect, showing antagonism at IC10 (CI = 9.93) and IC50 (CI = 1.67) but achieving synergy at IC90 (CI = 0.33). This result suggests that higher doses are necessary for synergistic activity, which may be crucial for determining optimal dosing strategies in combination therapy. In contrast, TENP + IMTB performs poorly, showing consistent antagonism across all inhibition levels (IC10: 3.47, IC50: 1.35, IC90: 1.38), indicating that this combination may be less effective and require further dose optimization. Comparisons between sunitinib (SNTB) and imatinib (IMTB) combinations reveal that IMTB combinations generally produce more consistent synergy, especially with ME and CVE-based formulations. For example, ME + IMTB exhibits strong synergy with CI values of 0.51 (IC10), 0.51 (IC50), and 0.60 (IC90), indicating stable cooperative effects across a wide range of doses. Meanwhile, MENP + IMTB shows synergy at low and medium inhibition levels (IC10: 0.65, IC50: 1.17) but antagonism at high inhibition (IC90: 2.12), suggesting a threshold effect where higher doses reduce efficacy. The overall findings emphasize that NP formulations substantially enhance drug potency and synergistic potential, with CVE-NP-based combinations demonstrating the most consistent synergy, particularly with SNTB and IMTB. Additionally, the data highlight that sunitinib-based combinations tend to produce dose-dependent effects, whereas imatinib-based combinations maintain more stable synergy across multiple inhibition levels. Consequently, future studies should focus on CVE-NP combinations and explore dosing adjustments for TE-NP and MENP to maximize synergistic effects and minimize antagonism.

### 3.4. Antitumor Effects of AgNPs Combined with Sunitinib and Imatinib: Mechanisms and Recent Literature Insights

**Figure 9:**
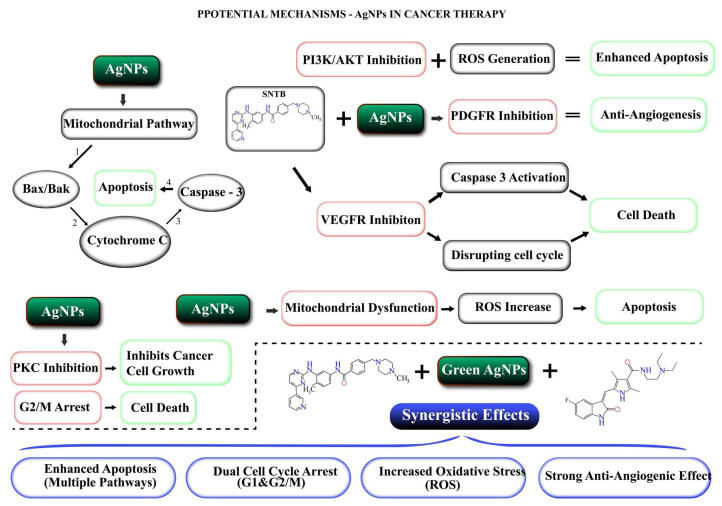
Potential mechanisms of AgNPs in cancer therapy: AgNPs induce apoptosis via the mitochondrial pathway by promoting cytochrome c release and caspase-3 activation. They also generate reactive oxygen species (ROS), causing oxidative stress and enhancing cell death. Additionally, AgNPs inhibit survival pathways, including PI3K/AKT and PDGFR, and disrupt angiogenesis through VEGFR inhibition, especially when combined with SNTB. Cell cycle arrest is induced at both G1/G2 and G2/M phases, inhibiting tumor proliferation. The combination of green-synthesized AgNPs with other agents exhibits synergistic effects, enhancing apoptosis, increasing ROS levels, and promoting antiangiogenic activity.

The current literature and the mechanistic insights represented in Figure 9 highlight the key mechanisms underlying the synergistic effects of silver nanoparticles (AgNPs) in combination with sunitinib or imatinib in cancer therapy. These mechanisms encompass oxidative stress induction, apoptosis promotion, cell cycle disruption, inhibition of pro-survival pathways, and antiangiogenic activity.

Enhanced ROS production and oxidative stress: AgNPs promote ROS generation, disrupting redox homeostasis and causing oxidative DNA, lipid, and protein damage, a key mechanism of cytotoxicity [49]. The combination with sunitinib or imatinib, known for inducing mitochondrial dysfunction, can amplify ROS production, driving cancer cell apoptosis via multiple pathways [58].

Apoptosis induction via caspase activation: AgNPs activate proapoptotic genes (e.g., p53 upregulated modulator of apoptosis-PUMA) and upregulate caspases 3, 8, and 9 [59]. Sunitinib and imatinib enhance this process by blocking survival signals through PDGFR and BCR-ABL inhibition, accelerating apoptosis through the mitochondrial pathway [60]

Dual cell cycle arrest (resting phase—Gap 1-G0/G1 and Gap2—mitosis phase—G2/M phases): AgNPs disrupt the G0/G1 phase by activating p53 and checkpoint inhibitors [61]. Concurrently, sunitinib and imatinib inhibit tyrosine kinase pathways (vascular endothelial growth factor receptor—VEGFR, platelet-derived growth factor receptor pathways—PDGFR, fusion protein—breakpoint cluster region BCR-ABL, halting the Gap2-Mitose- G2/M phase) [62]. The dual blockade prevents tumor proliferation and induces cell death.

Inhibition of prosurvival signaling pathways: AgNPs suppress Phosphoinositide 3-kinase/Izozime AKT (PI3K/Akt) and mitogen-activated protein kinase-MAPK pathways, reducing cancer cell viability [63]. Sunitinib and imatinib synergize by disrupting parallel survival signals through tyrosine kinase inhibition, further impairing tumor growth.

Antiangiogenic synergy: both AgNPs and sunitinib reduce VEGF expression, limiting tumor vascularization [64]. Their combined action significantly impairs the tumor’s ability to sustain growth through blood vessel formation.

## 4. Conclusions

UV-VIS spectrometry confirmed the formation of silver nanoparticles (AgNPs) from *Melissa officinalis* (ME AgNPs) and *Clematis vitalba* (CVE AgNPs). DLS (hydrodynamic diameter) and SEM analyses showed smaller particles for CVE AgNPs (11.1 ± 1.9 nm) than ME AgNPs (14.5 ± 2.9 nm). TE AgNPs 5% ETOH demonstrated the highest colloidal stability over time, maintaining consistent size and zeta potential.

From the experiments, it was confirmed that the coadministration of sunitinib and silver nanoparticles improved HUVEC cell viability, suggesting a protective effect against toxicity. All compounds exhibited antitumoral potential, with their combination with silver nanoparticles eliciting varying degrees of success:

TE AgNPs: exhibited strong cytotoxicity on HepG2 cells and enhanced sunitinib stability while reducing toxicity on HUVEC cells; ME AgNPs: showed potent cytotoxicity, with a synergistic effect when combined with sunitinib or imatinib; CVE AgNPs: displayed the highest cytotoxic effect, further amplified in combination therapies.

AgNPs-based treatments, particularly CVE extract and TE AgNPs 5% ETOH, achieved superior tumor selectivity. ME AgNPs combined with sunitinib or imatinib maintained strong efficacy and selectivity over 48 h. Antitumor mechanisms included ROS generation, caspase activation, dual-phase cell cycle arrest, and inhibition of PI3K/AKT and VEGFR/PDGFR. Synergistic effects with sunitinib and imatinib may enhance these mechanisms.

TE, ME, and CVE AgNPs, particularly in combination with sunitinib or imatinib, showed significant synergistic antitumor potential. TE AgNPs demonstrated notable stability, highlighting their promise for further in vivo studies in liver cancer therapy.

Further in vivo investigations and mechanism-based studies will be essential to validate these findings and determine their clinical applicability.

Although cancer as a disease remains a massive challenge for future generations of researchers, real therapeutic progress can be seen. Of the recent advances in drug therapy, the tyrosine kinase inhibitors have arguably been the most innovative advances, and their combination therapies with plant extract silver nanoparticles could bring significant insights into treatment antitumoral options.

Future research should focus on elucidating the specific mechanisms underlying the enhanced efficacy of these extracts and their respective nanoparticles. Furthermore, clinical studies will be necessary to assess the feasibility of integrating these natural products into existing cancer treatment regimens, potentially revolutionizing the approach to chemotherapy by minimizing side effects and improving patient outcomes.

## Figures and Tables

**Table 1:** *Taraxaci* extractum (TE) treatment groups.

TE 30% ETOHExtract—dandelion 30% ehtanolic extract (*Taraxaci extractum*);
TE AgNPs 5% ETOH—dandelion silver nanoparticles 5% ethanolic extract;
SNTB + TE 30% ETOH—sunitinib + dandelion 30% ehtanolic extract;
IMTB + TE 30% ETOH—imatinib + dandelion 30% ehtanolic extract;
SNTB + TE AgNPs 5% ETOH—sunitinib + dandelion silver nanoparticles 5% ehtanolic extract;
IMTB + TE AgNPs 5% ETOH—imatinib + dandelion silver nanoparticles 5% ehtanolic extract.

**Table 2:** *Melissae extractum* (ME) treatment groups.

ME 30% ETOHExtract—lemon balm 30% ehtanolic extract (*Melissae extractum*);
ME AgNPs 3% ETOH—lemon balm silver nanoparticles 3% ethanolic extract;
SNTB + ME 30% ETOH—sunitinib + lemon balm 30% ehtanolic extract;
IMTB + ME 30% ETOH—imatinib + lemon balm 30% ehtanolic extract;
SNTB + ME AgNPs 3% ETOH—sunitinib + lemon balm silver nanoparticles 3% ehtanolic extract;
IMTB + ME AgNPs 3% ETOH—imatinib + lemon balm silver nanoparticles 3% ehtanolic extract.

**Table 3:** *Clematis vitalbae extractum* (CVE) treatment groups.

CVE Extract—traveller’s joy aqueous extract (*Clematis vitalbae extractum*);
CVE AgNPs—*Clematis vitalbae extractum* silver nanoparticles;
SNTB + CVE AgNPs—sunitinib + *Clematis vitalbae extractum* silver nanoparticles;
IMTB + CVE AgNPs—imatinib + *Clematis vitalbae extractum* silver nanoparticles.

**Table 4:** Control groups.

SNTB—sunitinib (synthetic anticancer drug);
IMTB—imatinib (synthetic anticancer drug).

**Table 5:** The nanoparticle size of samples CVE AgNPs and ME AgNPs.

Sample	Nanoparticle Diameter (nm)		
	Min–Max	FWHM	Mean ± SD
CVE AgNPs	6–18	9–13	11.1 ± 1.9
ME AgNPs	9–22	11–17	14.5 ± 2.9

CVE AgNPs: *Clematis vitalbae extractum* silver nanoparticles; ME AgNPs: *Melissae extractum* silver nanoparticles.

**Table 6:** Hydrodynamic average diameter, PDI and zeta potential of green nanoparticles.

Sample	PDI *	NPs Hydrodynamic Size (nm)	Zeta Potential (mV)
24 h			
ME AgNPs 3% ETOH	0.200	940	−31.04
TE AgNPs 5% ETOH	0.248	218.7	−39.54
CVE AgNPs aqueous	1.588	5000	−34.05
48 h			
ME AgNPs 3% ETOH	0.171	362.2	−35.61
TE AgNPs 5% ETOH	0.249	209.3	−38.15
CVE AgNPs aqueous	0.814	2000	−34.60
72 h			
ME AgNPs 3% ETOH	0.247	581.4	−29.76
TE AgNPs 5% ETOH	0.291	243,9	−31.25
CVE AgNPs aqueous	0.564	4000	−27.54
One month			
ME AgNPs 3% ETOH	0.240	143	−12.29
TE AgNPs 5% ETOH [32]	0.179	200	−35.20
CVE AgNPs aqueous	0.179	507	−0.60

* Polydispersity Index (PDI) for formulations. A PDI ≤ 0.1 indicates a monodisperse system, where particles have uniform sizes. A PDI between 0.1 and 0.3 represents a slightly polydisperse system, which is characteristic of well-formulated colloidal dispersions. A PDI between 0.3 and 0.5 suggests a moderately polydisperse system, with greater variability in particle size distribution. A PDI ≥ 0.5 indicates a highly polydisperse system, often associated with significant aggregation and broad particle size distribution, which may affect formulation stability.

**Table 7:** Statistical significance of the ME influence on tumor cell viability compared to the SNTB control.

(I) HepG2_Treatment_ME_vs_SNTB	(J) Control_Group	Mean Difference (J–I)	Std. Error	Sig. (*p* Value)
ME AgNPs 1:1	Control_SNTB	0.61259 *	0.08632	0.003
ME AgNPs 1:2	Control_SNTB	0.39760 *	0.07941	0.022
SNTB + ME AgNPs 1:1	Control_SNTB	0.49988 *	0.07853	0.002

* The mean difference is significant at the 0.05 level. The abbreviations are explained in Table 2.

**Table 8:** Statistical significance of the ME influence on tumor cell viability compared to the IMTB control.

(I) HepG2_Treatment_ME_vs_IMTB	(J) Control_Group	Mean Difference (J–I)	Std. Error	Sig. (*p* Value)
ME AgNPs 1:1	Control_IMTB	0.45994 *	0.10684	0.028
IMTB + ME AgNPs 1:1	Control_IMTB	0.37692 *	0.07202	0.036

* The mean difference is significant at the 0.05 level. The abbreviations are explained in Table 2.

**Table 9:** Statistical significance of the CVE influence on tumor cell viability compared to the SNTB control.

(I) HepG2_Treatment_CVE_vs_SNTB	(J) Control_Group	Mean Difference (J–I)	Std. Error	Sig. (*p* Value)
CVE 1:1	Control_SNTB	0.51588 *	0.07305	0.004
CVE 1:2	Control_SNTB	0.46707 *	0.06980	0.010
CVE 1:4	Control_SNTB	0.40876 *	0.09289	0.035
CVE AgNPs 1:1	Control_SNTB	0.68835 *	0.06493	0.005
CVE AgNPs 1:2	Control_SNTB	0.50431 *	0.08197	0.005
SNTB + CVE AgNPs 1:1	Control_SNTB	0.37086 *	0.04998	0.000
SNTB + CVE AgNPs 1:2	Control_SNTB	0.26627 *	0.05312	0.018

* The mean difference is significant at the 0.05 level. The abbreviations are explained in Table 3.

**Table 10:** Statistical significance of the CVE influence on tumor cell viability compared to the IMTB control.

(I) HepG2_Treatment_CVE_vs_IMTB	(J) Control_Group	Mean Difference (J–I)	Std. Error	Sig. (*p* Value)
CVE 1:1	Control_IMTB	0.43148 *	0.08789	0.023
CVE 1:2	Control_IMTB	0.37713 ^†^	0.08879	0.066
CVE AgNPs 1:1	Control_IMTB	0.62611 *	0.06099	0.011
CVE AgNPs 1:2	Control_IMTB	0.42165 *	0.09653	0.028
IMTB + CVE AgNPs 1:1	Control_IMTB	0.40090 *	0.05021	0.001

* The mean difference is significant at the 0.05 level. ^†^ The mean difference is on the edge of significance. The abbreviations are explained in Table 3.

**Table 11:** IC50 values for tested compound on HUVEC and HepG2.

COMPOUND	IC50 (µg/mL)			
	HUVEC		HepG2	
	24 h	48 h	24 h	48 h
TE 30% ETOHExtract	12,757.22 ± 11,832.83	16,454.15 ± 9213.11	5874.16 ± 593.80	30,530.13 ± 36,511.17
TE AgNPs 5% ETOH	4.87 ± 1.77	5.91 ± 4.20	1.91 ± 0.30	1.11 ± 2.42
ME 30% ETOHExtract	20,693.57 ± 10,867.49	23,879.18 ± 11,180.72	29,077.68 ± 36,206.09	25,208.79 ± 11,539.90
ME AgNPs 3% ETOH	0.76 ± 0.72	1.04 ± 0.30	7.44 ± 4.70	7.21 ± 4.32
CVE Extract	9345.40 ± 4155.15	9345.41 ± 4155.15	2089.83 ± 2287.86	7932.39 ± 5859.23
CVE AgNPs	5.58 ± 6.97	5.58 ± 6.97	5.96 ± 3.11	5.54 ± 3.54
SNTB	252.48 ± 357.14	247.09 ± 339.23	134.48 ± 97.48	142.50 ± 112.80
IMTB	165.37 ± 90.76	171.07 ± 108.15	44.20 ± 5.00	44.99 ± 5.49
SNTB + TE 30% ETOH	11,290.90 ± 9226.68	12,440.12 ± 11,447.12	6586.45 ± 3154.23	6373.19 ± 2859.54
SNTB + TE AgNPs 5% ETOH	32.26 ± 10.23	31.11 ± 9.27	34.34 ± 18.95	40.58 ± 20.52
IMTB + TE 30% ETOH	2847.42 ± 1264.79	3754.29 ± 993.94	4201.78 ± 242.58	3101.41 ± 646.43
IMTB + TE AgNPs 5% ETOH	20.92 ± 0.66	19.61 ± 1.67	22.46 ± 1.73	22.68 ± 4.13
SNTB + ME 30% ETOH	38,239.95 ± 68,477.77	6091.39 ± 1211.48	21,323.24 ± 19,207.04	19,279.42 ± 13,526.20
SNTB + ME AgNPs 3% ETOH	108.86 ± 61.74	114.07 ± 58.69	71.61 ± 46.60	55.92 ± 42.69
IMTB + ME 30% ETOH	8830.77 ± 1193.40	9625.93 ± 1299.08	8179.60 ± 2347.55	4030.21 ± 1925.17
IMTB + ME AgNPs 3% ETOH	67.71 ± 26.90	60.88 ± 20.97	39.72 ± 5.47	35.47 ± 13.86
SNTB + CVE AgNPs	106.09 ± 114.21	57.60 ± 24.67	62.22 ± 34.39	57.60 ± 24.67
IMTB + CVE AgNPs	96.68 ± 51.42	22.58 ± 8.71	37.19 ± 10.84	22.58 ± 8.71

## Data Availability

The original contributions presented in the study are included in the article/Supplementary Material; further inquiries can be directed to the corresponding author.

## Supplementary Materials

The following supporting information can be downloaded at: https://www.mdpi.com/article/10.3390/biomedicines13030641/s1, Figure S1: (a) DLS measurements of CVE AgNPs; (b) SEM images of CVE AgNPs; (c) Zeta potential measurements for CVE AgNPs; Figure S2: Normality histogram for TE groups vs. Sunitinib control group on HepG2 cells; Figure S3: Normal Q-Q plot for TE groups vs. Sunitinib control group on HepG2 cells; Figure S4: Normality histogram for TE groups vs. Imaitinib control group on HepG2 cells; Figure S5: Normal Q-Q plot for TE groups vs. Imatinib control group on HepG2 cells; Figure S6: Cell viability (%) of HUVEC lines after: (a) 24 h of treatment with various dilutions of Taraxaci extractum—based samples and the chemotherapeutic drugs Sunitinib and Imatinib and (b) presents the same measurements after 48 h of treatment; Figure S7: Normality histogram for ME groups vs. Sunitinib control group on HepG2 cells; Figure S8: Normal Q-Q plot for ME groups vs. Sunitinib control group on HepG2 cells; Figure S9: Normality histogram for ME groups vs. Imatinib control group on HepG2 cells; Figure S10: Normal Q-Q plot for ME groups vs. Imatinib control group on HepG2 cells; Figure S11: Cell viability (%) of HUVEC lines after: (a) 24 h of treatment with various dilutions of Melissae extractum—based samples and the chemotherapeutic drugs Sunitinib and Imatinib and (b) presents the same measurements after 48 h of treatment; Figure S12: Normality histogram for CVE groups vs. Sunitinib control group on HepG2 cells; Figure S13: Normal Q-Q plot for CVE groups vs. Sunitinib control group on HepG2 cells; Figure S14: Normality histogram for CVE groups vs. Imatinib control group on HepG2 cells; Figure S15: Normal Q-Q plot for CVE groups vs. Imatinib control group on HepG2 cells; Table S1. Dose-effect parameters and CI values for compound combinations.

## Abbreviations

The following abbreviations are used in this manuscript:

TE	*Taraxaci extractum*
ME	*Melissae extractum*
CVE	*Clematis vitalbae extractum*
SNTB	Sunitinib
IMTB	Imatinib
HepG2	Human hepatocellular carcinoma cell line
HUVEC	Human umbilical vein endothelial cells
AgNPs	Silver nanoparticles
DLS	Dynamic light scattering
ELS	Electrophoretic light scattering
SEM	Scanning electron microscopy
FWHM	Full width at half maximum
ROS	Reactive oxygen species
DNA	Deoxyribonucleic acid
Bax/Bak	Bcl-2-associated X protein/Bcl-2 antagonist killer
PI3K/AKT	Phosphoinositide 3-kinase/Protein kinase B
PDGFR	Platelet-derived growth factor receptor
VEGFR	Vascular endothelial growth factor receptor
PKC	Protein Kinase C
