# Supplementary material for: Synergistic Effects of Green Nanoparticles on Antitumor Drug Efficacy in Hepatocellular Cancer
Source: Biomedicines. 2025 Mar 5;13(3):641. doi: 10.3390/biomedicines13030641 (PMC11940350; doi:10.3390/biomedicines13030641)
Supplement: Supplementary file 1 [file biomedicines-13-00641-s001.zip › biomedicines-3460614-supplementary.pdf]

Supplementary Information

Article

Synergistic Effects of Green Nanoparticles on Antitumor Drug Efficacy in Hepatocellular Cancer

Intensity Distribution | ACF

(a)

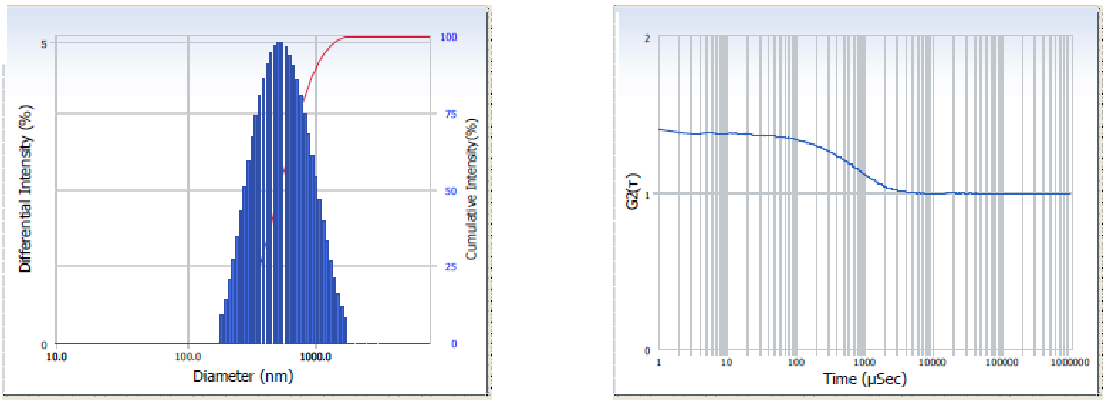

Distribution Results (Contin)

Peak	Diameter (nm)	Std. Dev.
1	633.2	316.0
2	0.0	0.0
3	0.0	0.0
4	0.0	0.0
5	0.0	0.0
Average	633.2	316.0

Residual : 1.388e-003 (O.K)

Cumulants Results

Diameter	(d)	: 507.1	(nm)
Polydispersity Index	(P.I.)	: 0.179	
Diffusion Const.	(D)	: 9.726e-009	(cm^2/sec)
Molecular Weight		: 1.057e+010	

Measurement Condition

Temperature	: 25.1	(°C)
Diluent Name	: WATER	
Refractive Index	: 1.3328	
Viscosity	: 0.8858	(cP)
Scattering Intensity	: 30453	(cps)
Attenuator 1	: 0.65	(%)

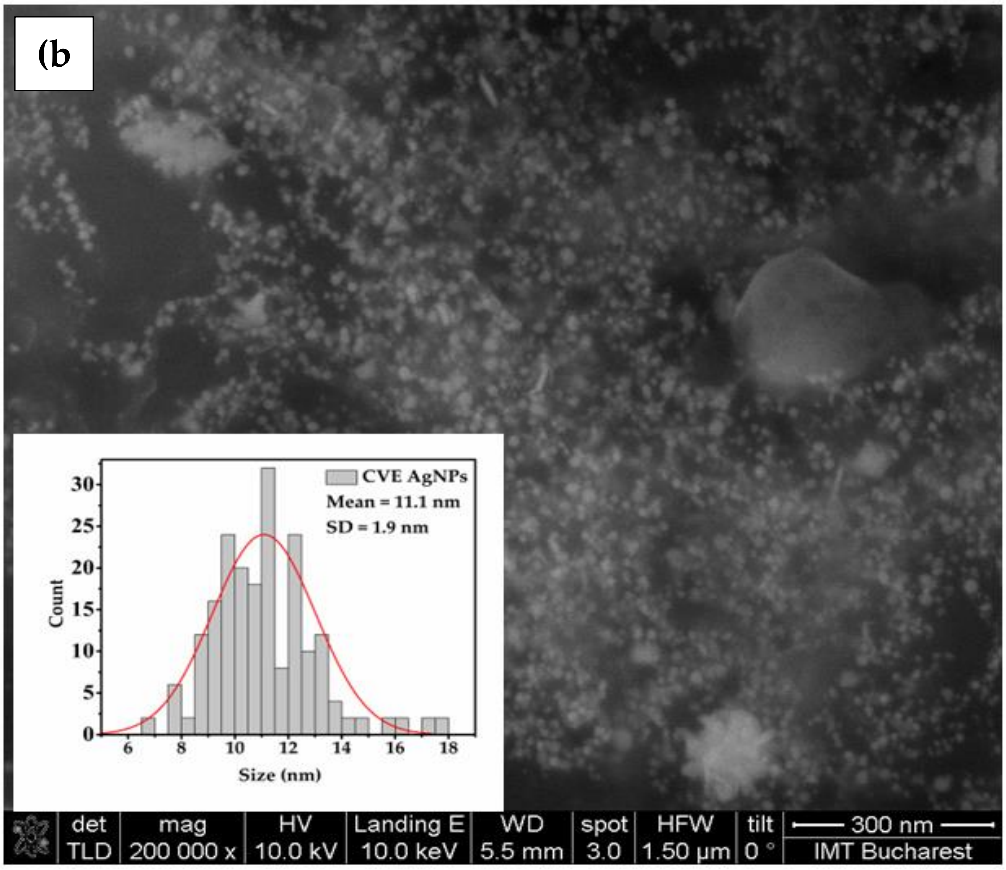

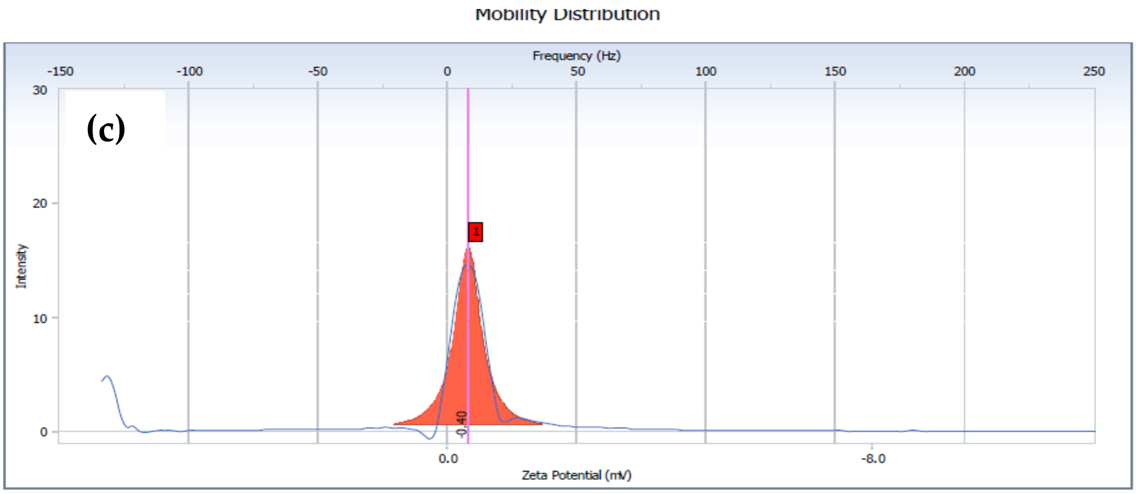

Measurement Results

Zeta Potential	: -0.40	(mV)		Doppler shift	: 8.14	(Hz)
Mobility	: -3.093e-006	(cm²/Vs)		Base Frequency	: 124.5	(Hz)
Conductivity	: 0.0339	(mS/cm)		Conversion Equation	: Smoluchowski	

Zeta Potential of Cell			Diluent Properties		
Upper Surface	: -0.60	(mV)	Diluent Name	: WATER	
Lower Surface	: -0.66	(mV)	Temperature	: 25.1	(°C)
Cell Condition			Refractive Index	: 1.3328	
Cell Type	: Flow Cell		Viscosity	: 0.8858	(cP)
Avg. Electric Field	: -545.06	(V/cm)	Dielectric Constant	: 78.2	
Avg. Current	: -0.92	(mA)			

Figure S1. (a) DLS measurements of CVE AgNPs; **(b)** SEM images of CVE AgNPs; **(c)** Zeta potential measurements for CVE AgNPs.

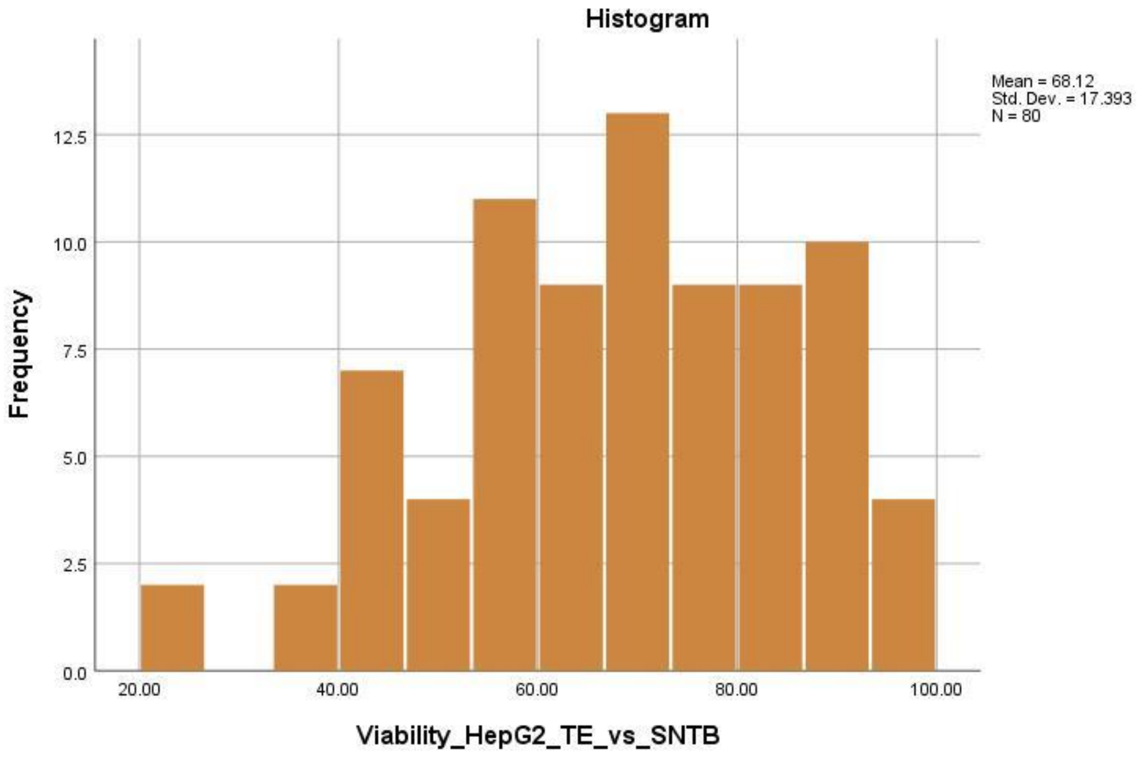

Figure S2. Normality histogram for TE groups vs Sunitinib control group on HepG2 cells.

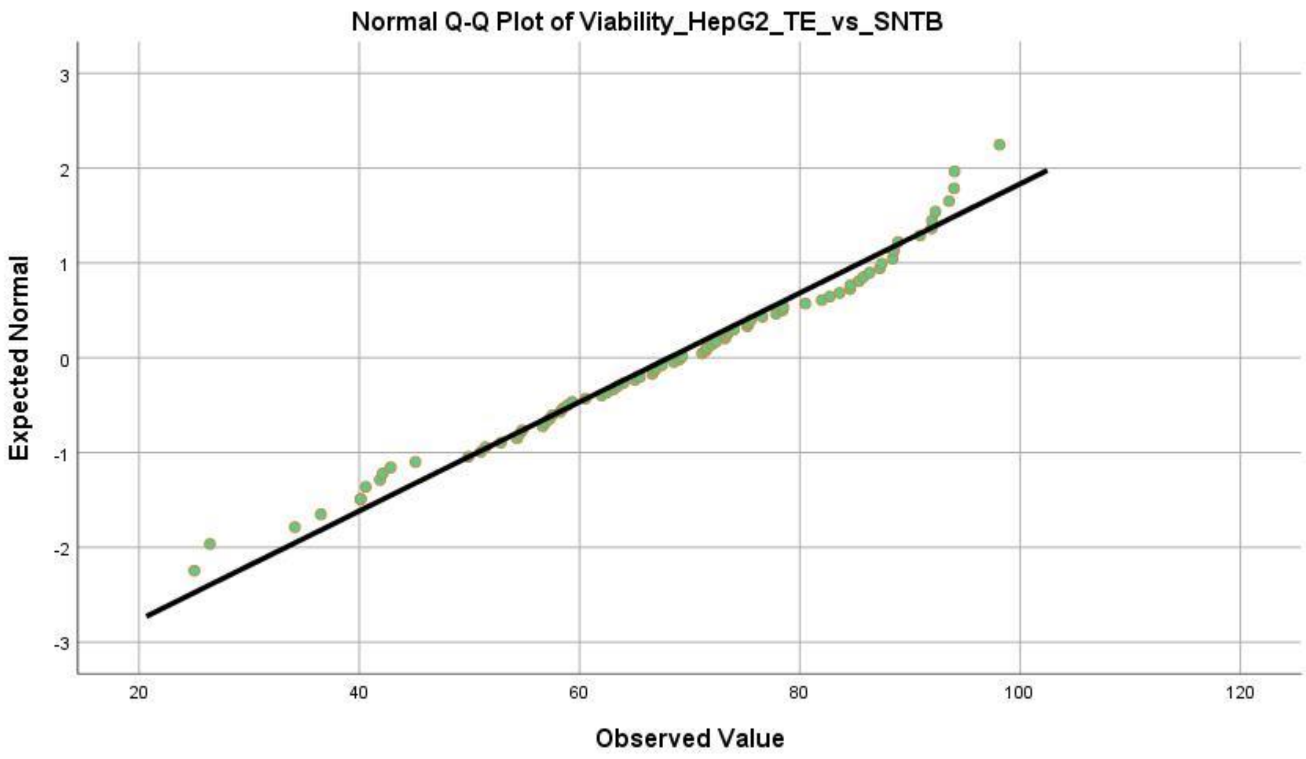

Figure S3. Normal Q-Q plot for TE groups vs Sunitinib control group on HepG2 cells.

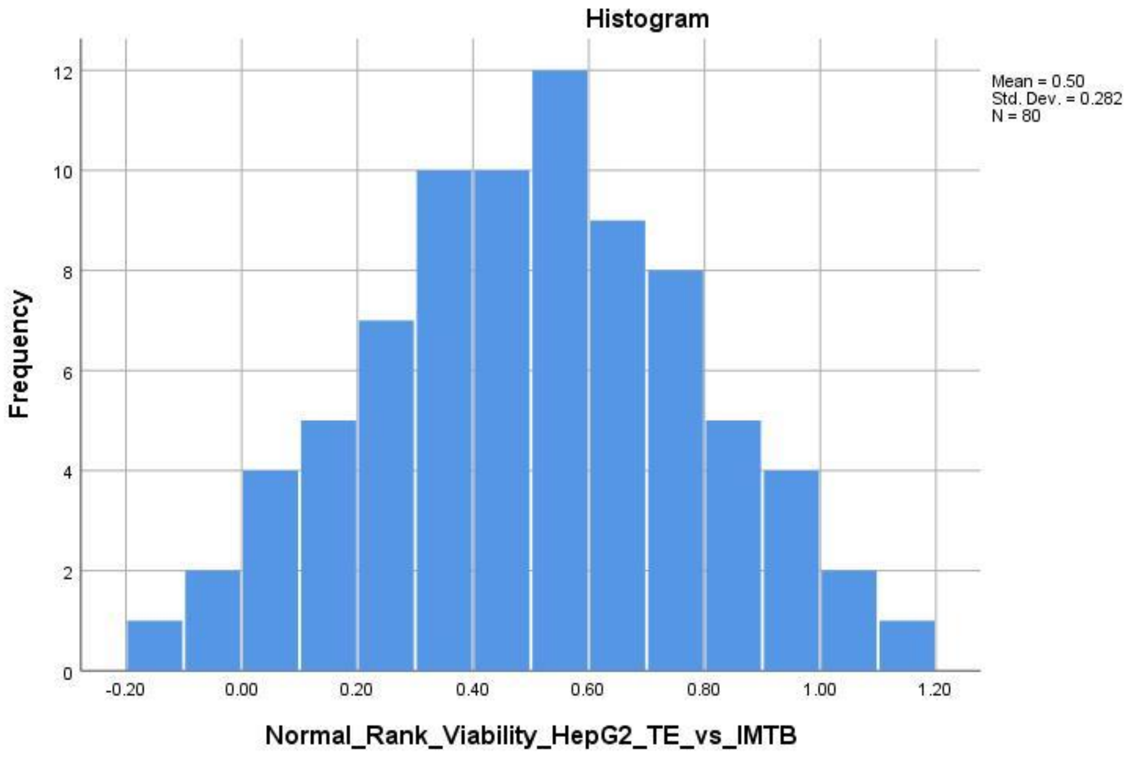

Figure S4. Normality histogram for TE groups vs Imaitinib control group on HepG2 cells.

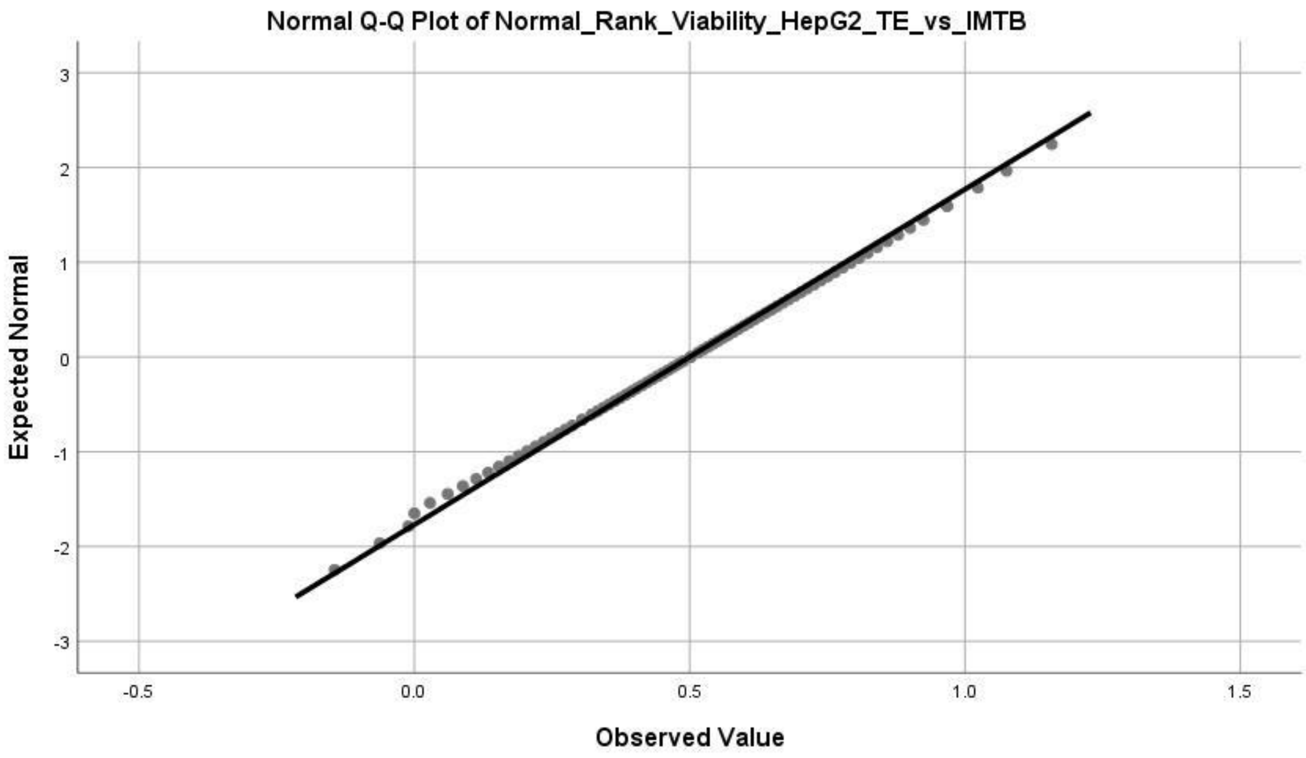

Figure S5. Normal Q-Q plot for TE groups vs Imatinib control group on HepG2 cells.

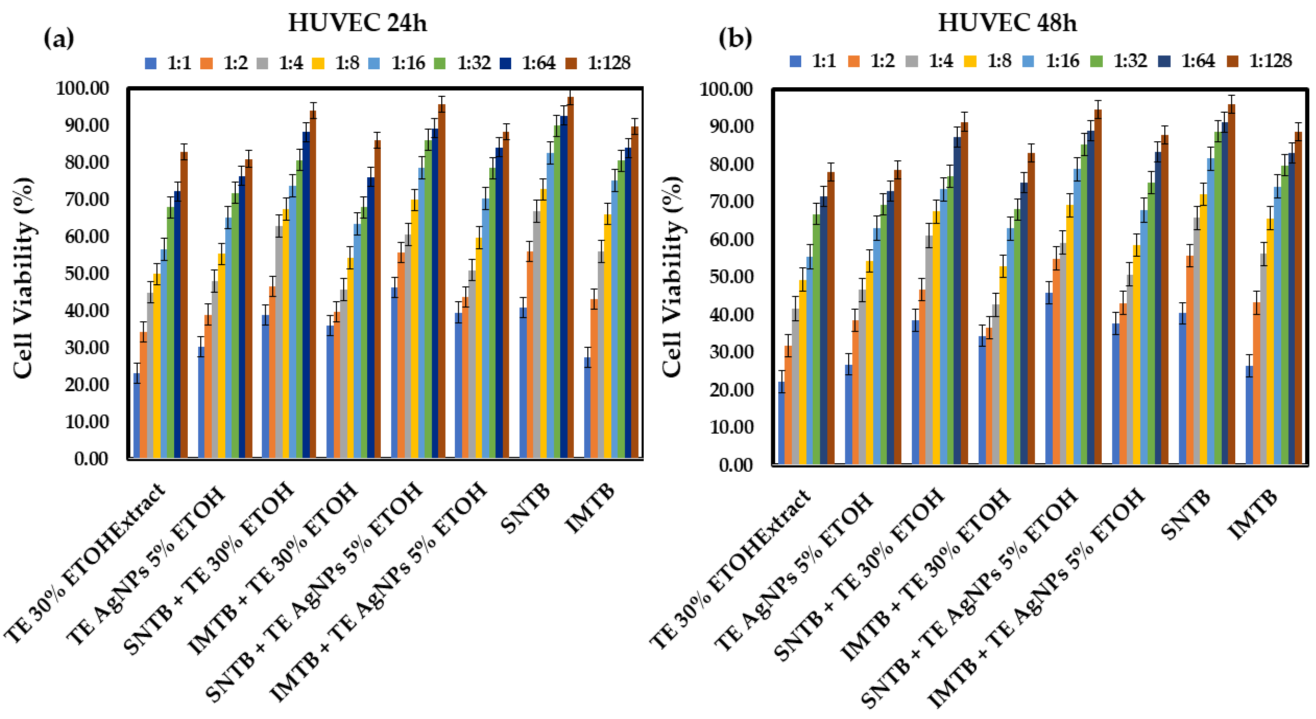

Figure S6. Cell viability (%) of HUVEC lines after: (a) 24 hours of treatment with various dilutions of *Taraxaci extractum* - based samples and the chemotherapeutic drugs Sunitinib and Imatinib and (b) presents the same measurements after 48 hours of treatment.

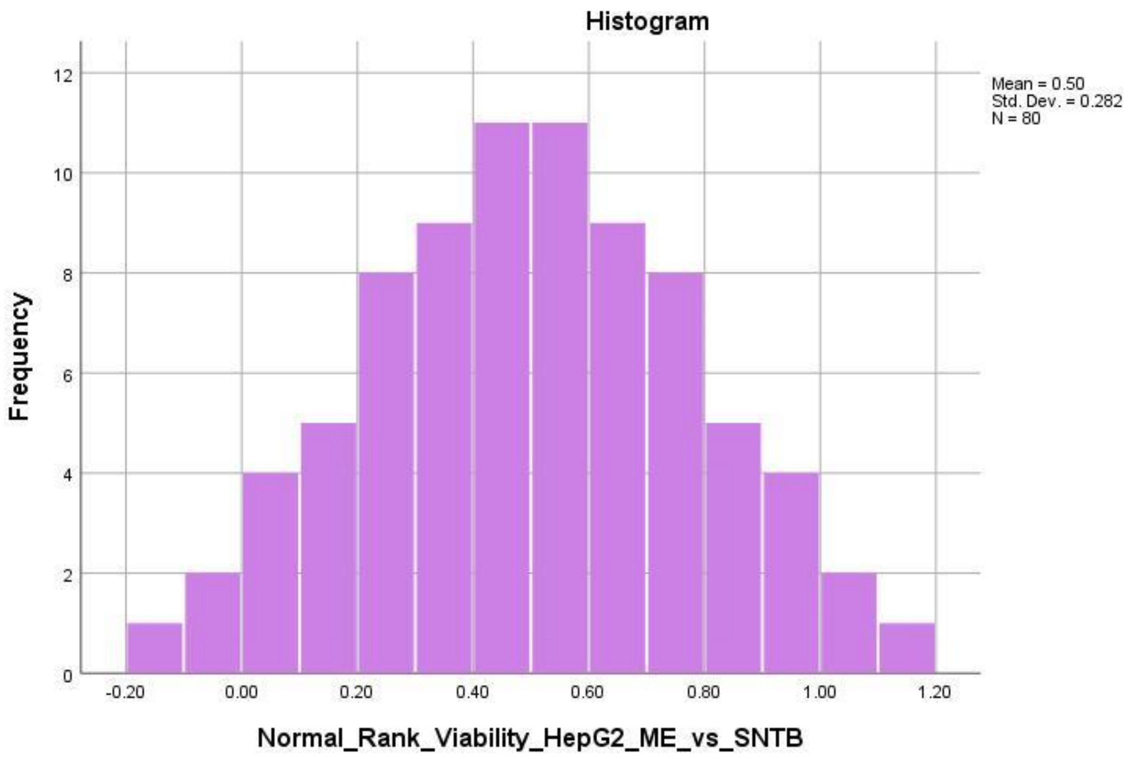

Figure S7. Normality histogram for ME groups vs Sunitinib control group on HepG2 cells.

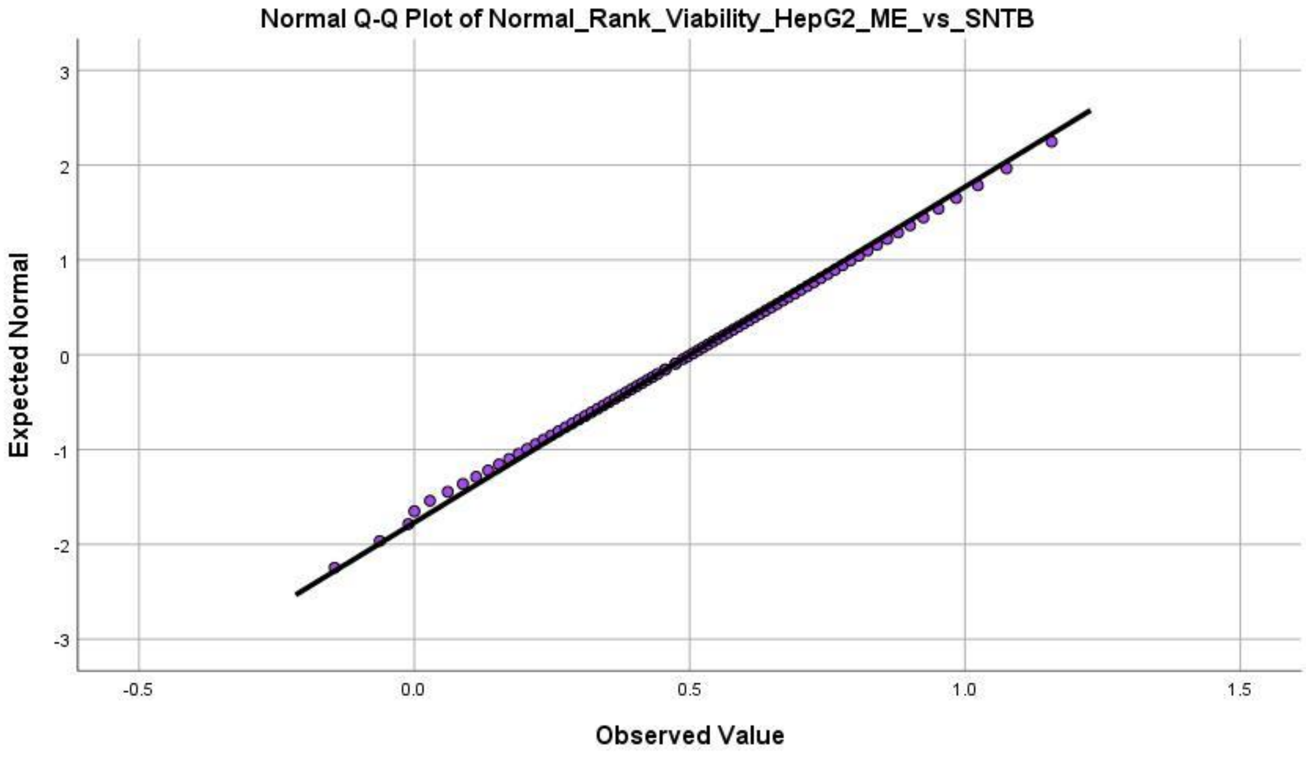

Figure S8. Normal Q-Q plot for ME groups vs Sunitinib control group on HepG2 cells.

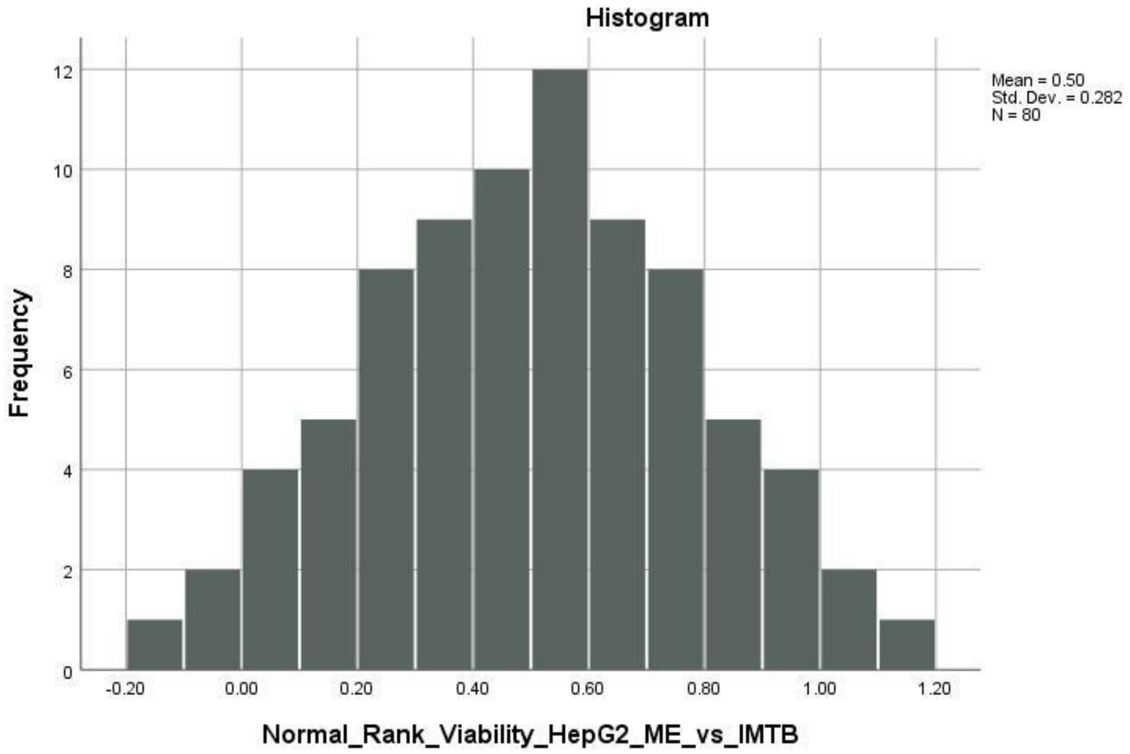

Figure S9. Normality histogram for ME groups vs Imatinib control group on HepG2 cells.

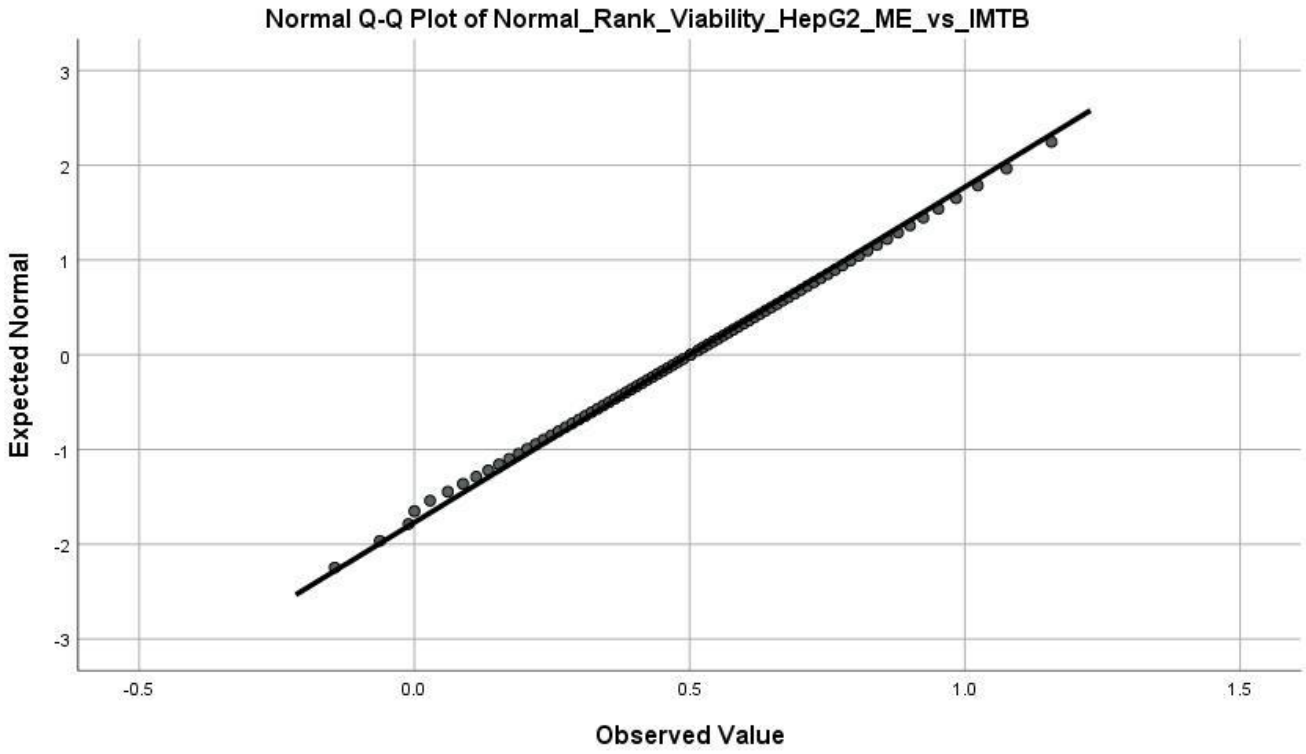

Figure S10. Normal Q-Q plot for ME groups vs Imatinib control group on HepG2 cells.

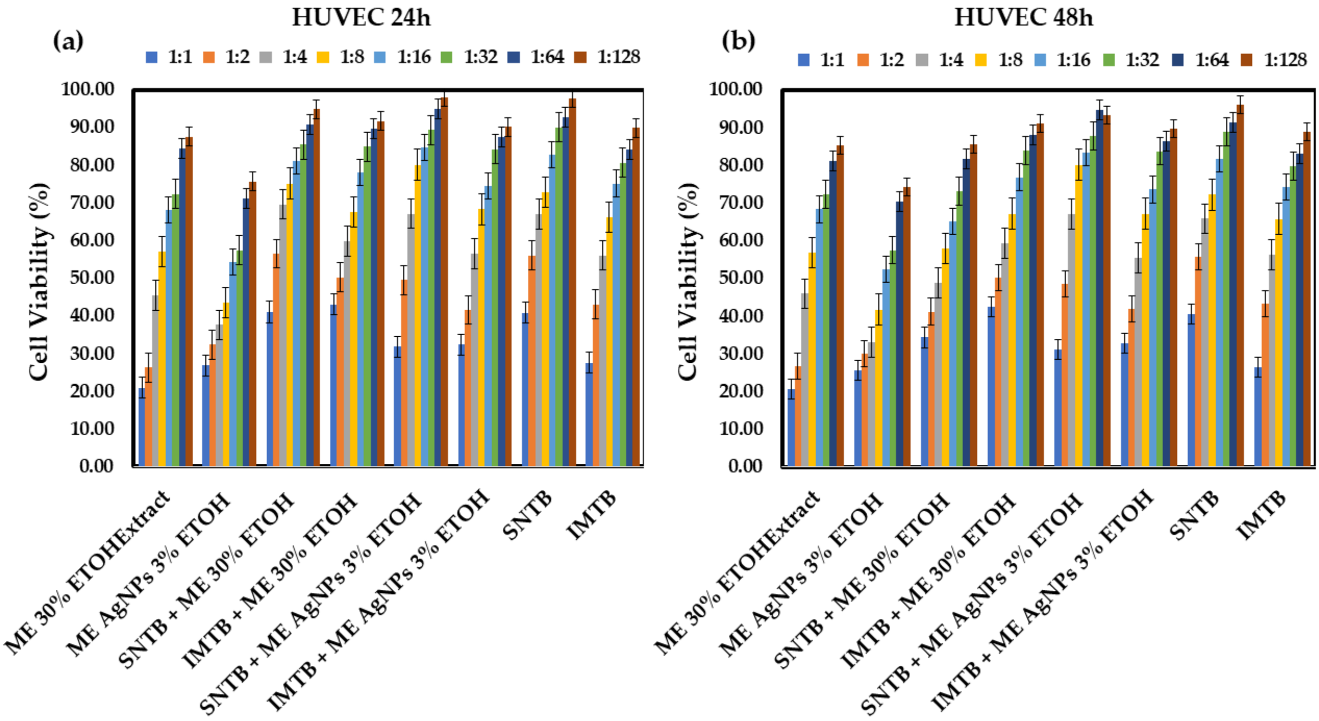

Figure S11. Cell viability (%) of HUVEC lines after: **(a)** 24 hours of treatment with various dilutions of *Melissae extractum* -based samples and the chemotherapeutic drugs Sunitinib and Imatinib and **(b)** presents the same measurements after 48 hours of treatment.

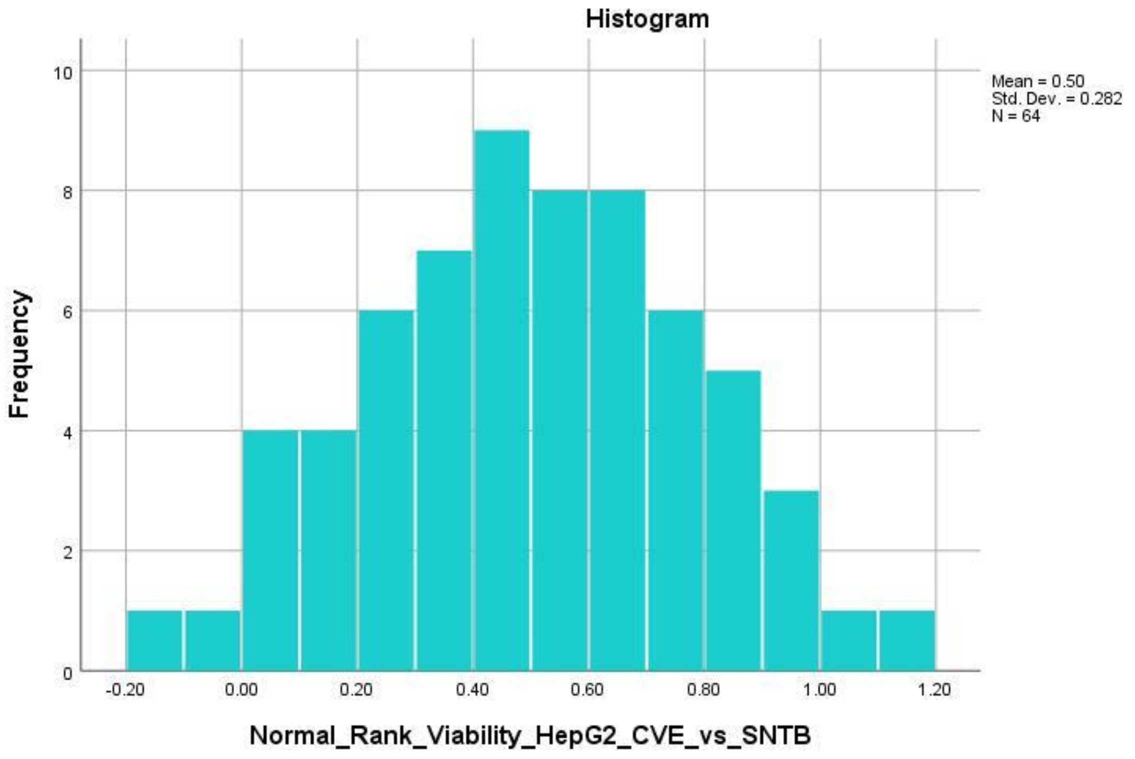

Figure S12. Normality histogram for CVE groups vs Sunitinib control group on HepG2 cells.

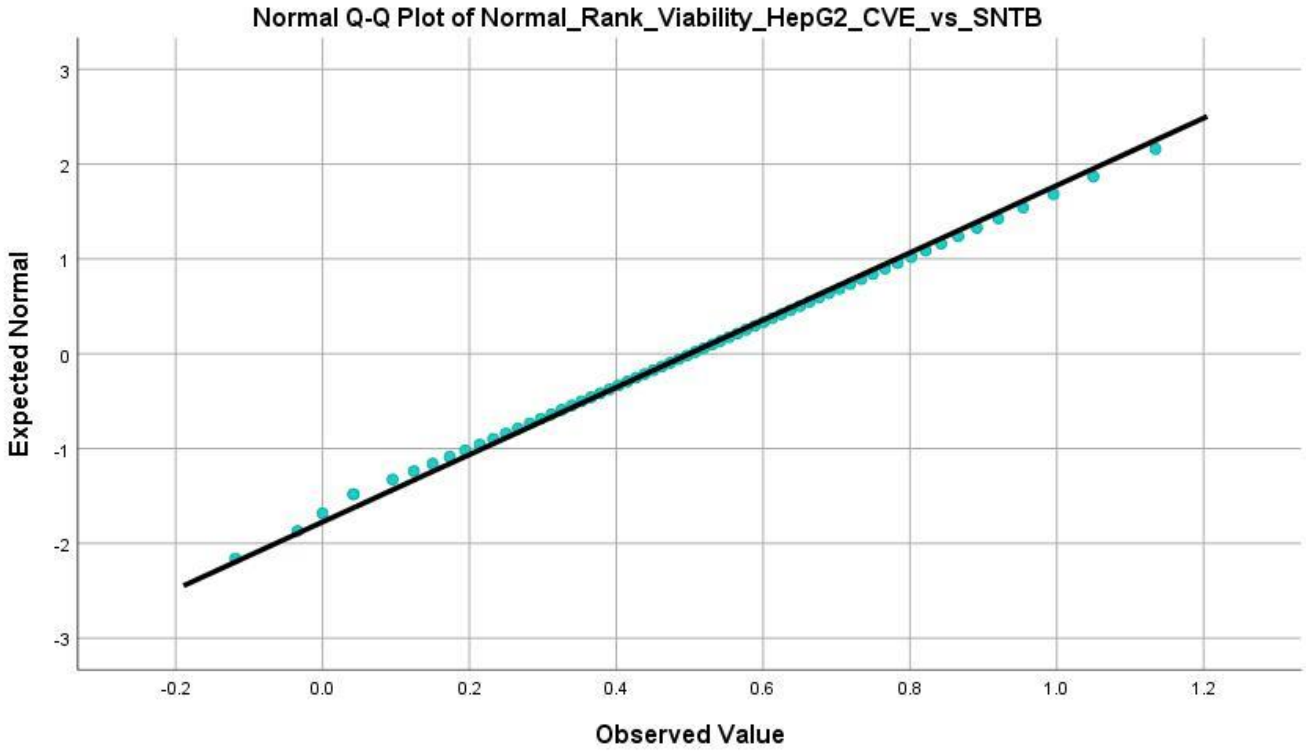

Figure S13. Normal Q-Q plot for CVE groups vs Sunitinib control group on HepG2 cells.

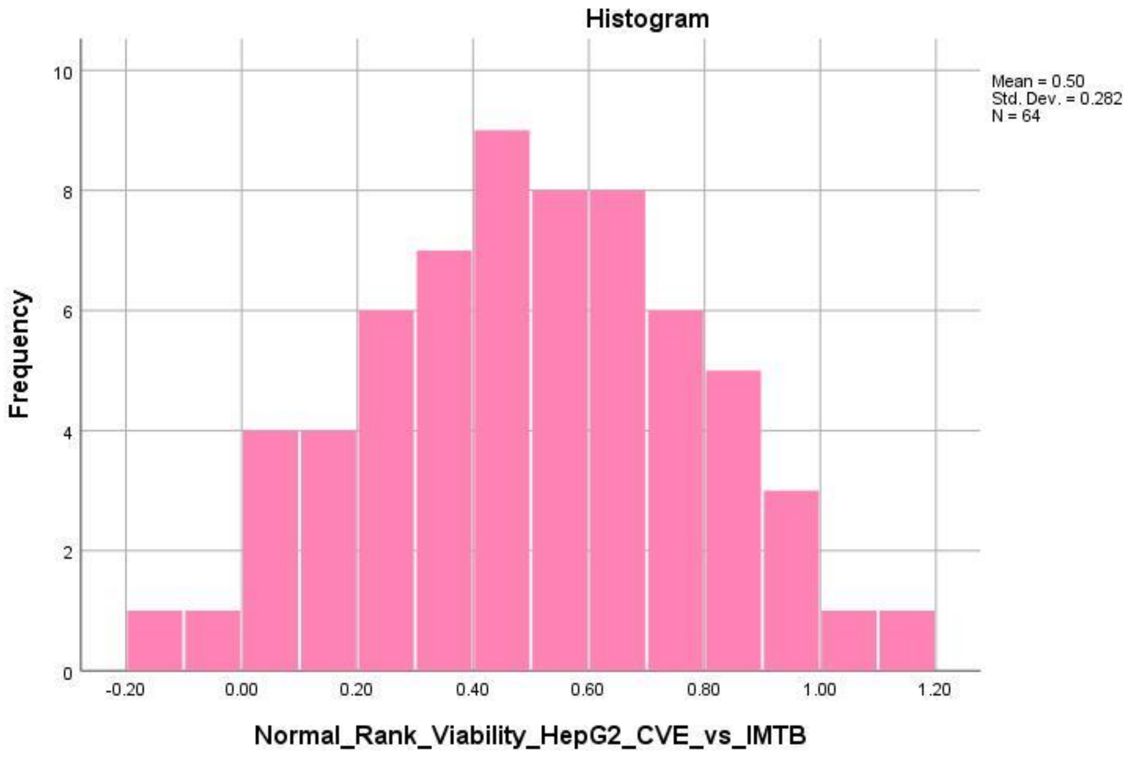

Figure S14. Normality histogram for CVE groups vs Imatinib control group on HepG2 cells.

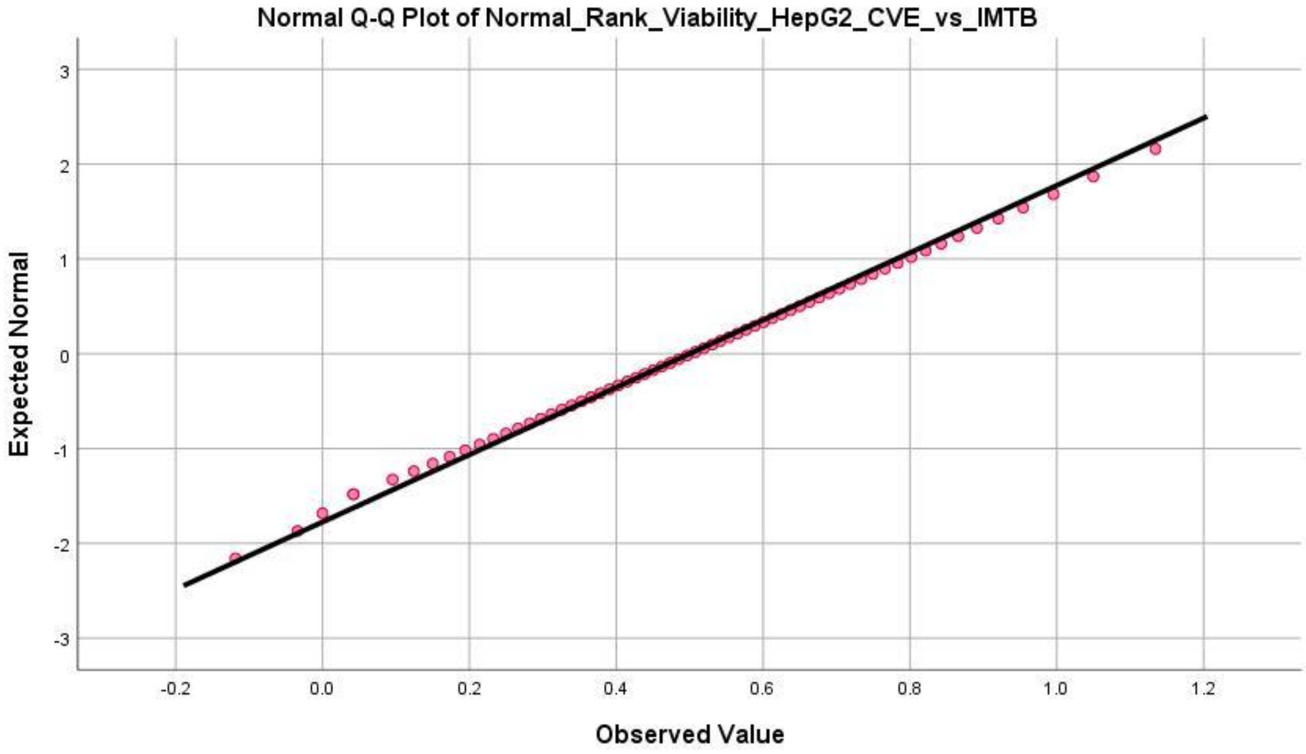

Figure S15. Normal Q-Q plot for CVE groups vs Imatinib control group on HepG2 cells.

Table S1. Dose-effect parameters and CI values for compound combinations

DRUG COMBO (24 H)	DOSE-EFFECT PARAMETERS			CI VALUES					
	Dm (µg/mL)	m	r	IC_{10}		IC_{50}		IC_{90}	
**TE**	5330.19232	0.71654	0.99901	-		-		-	
**TE-NP**	2.02456	0.52875	0.99674	-		-		-	
**ME**	99999.99999	0.50178	0.97907	-		-		-	
**ME-NP**	19.69696	0.85392	0.94933	-		-		-	
**CVE**	781.25000	0.43602	0.98852	-		-		-	
**CVE-NP**	9.10327	1.17636	0.96133	-		-		-	
**SNTB**	180.39938	0.80609	0.98972	-		-		-	
**IMTB**	45.52460	0.76166	0.99246	-		-		-	
**TE + SNTB**	6586.45765	0.74514	0.98748	1.50	*Ant*	1.37	*Ant*	1.27	*Ant*
**TENP + SNTB**	34.33745	0.70854	0.99024	2.22	*Ant*	0.91	*Add*	0.52	*Syn*
**ME + SNTB**	32097.99345	0.68980	0.99373	1.50	*Ant*	1.03	*Add*	1.22	*Ant*
**MENP + SNTB**	210.00000	0.72307	0.99026	1.13	*Ant*	1.62	*Ant*	2.32	*Ant*
**CVENP + SNTB**	81.89002	0.97169	0.97703	1.00	*Add*	0.89	*Syn*	0.96	*Add*
**TE + IMTB**	3697.32708	0.77543	0.99513	1.21	*Ant*	1.01	*Add*	0.85	*Syn*
**TENP + IMTB**	22.59409	0.69406	0.99679	1.65	*Ant*	0.95	*Add*	0.81	*Syn*
**ME + IMTB**	9012.37545	0.69959	0.99502	0.92	*Add*	0.88	*Syn*	1.04	*Add*
**MENP + IMTB**	46.88795	0.79574	0.99609	1.20	*Ant*	1.09	*Ant*	1.00	*Syn*
**CVENP + IMTB**	38.70985	1.02144	0.99096	1.84	*Ant*	1.03	*Add*	0.68	*Syn*

DRUG COMBO (48 H)	DOSE-EFFECT PARAMETERS			CI VALUES					
	Dm (µg/mL)	m	r	IC_{10}		IC_{50}		IC_{90}	
**TE**	100000.00000	0.33895	0.97745	-		-		-	
**TE-NP**	1.10937	0.41658	0.96545	-		-		-	
**ME**	25208.81592	0.55597	0.99868	-		-		-	
**ME-NP**	20.00000	0.77374	0.94893	-		-		-	
**CVE**	2367.46845	0.11950	0.98754	-		-		-	
**CVE-NP**	12.17240	0.88384	0.93159	-		-		-	
**SNTB**	400.00000	0.59189	0.99176	-		-		-	
**IMTB**	46.07692	0.71536	0.99087	-		-		-	
**TE + SNTB**	6373.19942	0.69189	0.99089	1.84	*Ant*	0.13	*Syn*	0.04	*Syn*
**TENP + SNTB**	40.57676	0.63762	0.99582	9.93	*Ant*	1.67	*Ant*	0.33	*Syn*
**ME + SNTB**	29097.01940	0.64749	0.99436	2.41	*Ant*	1.44	*Ant*	0.87	*Syn*
**MENP + SNTB**	180.34942	0.62625	0.98526	0.74	*Syn*	0.86	*Syn*	1.19	*Ant*
**CVENP + SNTB**	104.12826	0.76444	0.98556	0.87	*Syn*	0.69	*Syn*	0.76	*Syn*
**TE + IMTB**	2518.07930	0.78164	0.98904	1.27	*Ant*	0.24	*Syn*	0.17	*Syn*
**TENP + IMTB**	22.67786	0.55479	0.99909	3.47	*Ant*	1.35	*Ant*	1.38	*Ant*
**ME + IMTB**	4030.21478	0.63907	0.98907	0.51	*Syn*	0.51	*Syn*	0.60	*Syn*
**MENP + IMTB**	51.00452	0.60455	0.99260	0.65	*Syn*	1.17	*Ant*	2.12	*Ant*
**CVENP + IMTB**	29.44587	0.76188	0.98636	0.81	*Syn*	0.73	*Syn*	0.69	*Syn*
